# Microbial remediation of recalcitrant pollutants in soil and geo-environmental engineering systems

**DOI:** 10.3389/fbioe.2026.1904267

**Published:** 2026-07-15

**Authors:** Anjana S, A. K. Priya

**Affiliations:** 1 Division of Civil Engineering, Karunya Institute of Technology and Sciences, Coimbatore, Tamil Nadu, India; 2 Division of Biotechnology, Karunya Institute of Technology and Sciences, Coimbatore, Tamil Nadu, India; 3 Water Institute, A Centre of Excellence, Karunya Institute of Technology and Sciences, Coimbatore, Tamil Nadu, India

**Keywords:** biodegradation, geo-environmental engineering, heavy metals, MICP, microbial consortia, microbial remediation, recalcitrant pollutants, soil contamination

## Abstract

Polycyclic aromatic hydrocarbons (PAHs), petroleum hydrocarbons, chlorinated compounds, pesticides, pharmaceuticals, per- and polyfluoroalkyl substances and heavy metals are all examples of pollutants that are more difficult to degrade in the environment, are toxic, and persist in the environment for an extended period. Traditional remediation practices are often high-cost, high energy, and can have a negative impact on soil ecosystems. Microbial remediation has proven to be an emerging sustainable and eco-friendly technology using bacteria, fungi, actinomycetes, and microbial consortia to degrade, transform, immobilize, and/or detoxify contaminants through a variety of processes such as biodegradation, biosorption, bioaccumulation, biotransformation, biomineralization, co-metabolism, and biofilm-mediated processes. This review covers the types of pollutants that are recalcitrant, the microbial communities in the soil, and the most significant microbial processes associated with the remediation of recalcitrant pollutants. Additionally, the most important applications of geo-environmental engineering (GE) are critically discussed, including biostimulation, bioaugmentation, rhizoremediation, mycoremediation, microbially induced carbonate precipitation (MICP), and permeable reactive biobarriers. The factors affecting remediation effectiveness, advanced monitoring methods, existing challenges, and emerging innovations such as synthetic biology, engineered microbial consortia, nanobioremediation, and artificial intelligence are also pointed out. Microbial processes combined with geo-environmental engineering can be a promising way to restore the soil sustainably and to protect the environment for the long term.

## 1 Introduction

**FIGURE 1:**
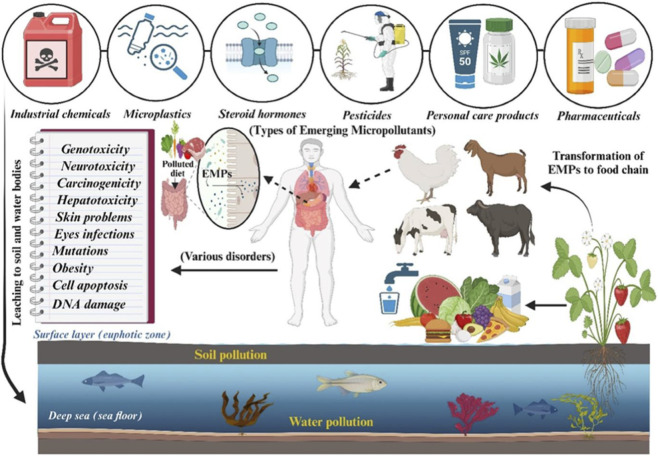
Major emerging micropollutants and their pathways of environmental exposure and health impacts (Ahmad et al., 2024).

Soil contamination has become a major environmental problem associated with industrialization, urbanization, mining, intensive agriculture, land overuse, and improper waste disposal. Contaminated soils can act as long-term reservoirs of hazardous chemicals, with consequences for ecosystem function, groundwater quality, crop production, food safety, and human health. The persistence, mobility, bioavailability, and ecological effects of recalcitrant contaminants therefore require remediation strategies that reduce not only parent compound concentrations, but also exposure, toxicity, and long-term environmental risk (Achal et al., 2011). Remediation of contaminated soils has become a necessity for the protection of the environment and sustainable use of land. Persistent contaminants pose a special threat amongst the different contaminants, as they do not readily break down in nature (DeJong et al., 2010). These pollutants have complex chemical structures, are toxic, and are difficult to biodegrade. They are able to persist in the soil for extended periods of time and are also known to bioaccumulate over time, creating long-term, harmful effects on ecosystems (Bisht et al., 2015). The persistence is primarily due to their physical and chemical characteristics such as hydrophobicity, high molecular weight, containing halogens, and chemical stability, making them less prone to natural degradation. A variety of organic and inorganic contaminants are considered to be recalcitrant pollutants in soils (Tyagi et al., 2011). These include polycyclic aromatic hydrocarbons (PAHs), petroleum hydrocarbons, chlorinated organic compounds, polychlorinated biphenyls (PCBs), pesticides/herbicides, synthetic dyes, pharmaceuticals, antibiotics, per- and polyfluoroalkyl substances (PFAS), and toxic heavy metals (Chakraborty et al., 2012). Figure 1 illustrates the major categories of emerging pollutants, their environmental pathways, and their potential impacts on ecosystems and human health. They come from several sources such as petrochemical industries, manufacturing facilities, mining, agricultural run-off, landfill leachates, wastewater effluent discharge, and accidental spills (Das and Chandran, 2011). Once in soil systems, they can change their environmental behaviour and risks through transport, absorption, transformation, or bioaccumulation. Recalcitrant pollutants can cause severe environmental and health issues if they are not removed (Gadd, 2010). A lot of these contaminants are known to cause cancer, mutations, birth defects, and disrupt endocrine systems. Furthermore, the build-up in soil may affect diversity, fertility, nutrient cycling, and ecosystem function (Sharma, 2020). Groundwater can also be contaminated and lead to further exposure risks to animals and humans when consumed through the food chain *via* plants. Therefore, effective cleaning technologies for these persistent pollutants are an important research focus. Methods for the treatment of contaminated soil include traditional techniques like excavation, land filling, thermal treatment, soil washing, chemical oxidation, and soil stabilization. These methods can be quite effective in the removal of contaminants, but they are also expensive, energy-intensive, potentially polluting, and can impact natural soil ecosystems. Moreover, these traditional techniques may not be suitable for contaminated sites with extensive contamination or mixed contaminants, which has led to the development of interest in sustainable and eco-friendly solutions (Azubuike et al., 2016). The treatment of contaminated soils using microbes has become a new and environmentally friendly technology. Many contaminants are very amenable to being used, transformed, immobilized, or detoxified by microorganisms. Various types of bacteria, fungi, archaea, and actinomycetes can degrade recalcitrant organic pollutants, including PAHs, petroleum hydrocarbons, and chlorinated compounds. In contrast, heavy metals and metalloids are not degraded but can be transformed, immobilized, or detoxified through microbial processes that alter their speciation, mobility, bioavailability, and toxicity. In natural and engineered approaches to clean polluted environments, microorganisms play a crucial role through processes such as biodegradation, biosorption, bioaccumulation, biotransformation, bio-mineralization, and co-metabolism (Ali et al., 2013). The recent development of environmental microbiology, molecular biology, omics technologies, and biotechnology has greatly enhanced the knowledge of the response of microbes to contamination. Incorporating microbial processes into geo-environmental engineering practices provides a new range of possibilities for sustainable site cleaning and environmental remediation, such as the use of microbial groups, genetically modified microorganisms, bioaugmentation, biostimulation, rhizoremediation and mycoremediation (Megharaj et al., 2011). Microbial remediation technologies are now being used in geo-environmental engineering solutions to remediate contaminated soil and groundwater. These techniques include permeable reactive biobarriers, bioelectrochemical systems, microbially induced carbonate precipitation (MICP), and biologically enhanced stabilization techniques (Patel et al., 2020). These practices can remove contaminants, enhance soil health, safeguard groundwater quality, and provide long-term environmental sustainability. The integration of microbial processes and engineering techniques is an important interdisciplinary research field. With the increasing environmental problems associated with persistent contaminants, knowledge of microbial remediation processes and applications is crucial. The objective of this review is to examine the occurrence and properties of persistent pollutants in soils and in geo-environmental systems, the various microbial processes which can remove pollutants, existing and emerging techniques for the remediation, the factors that affect the effectiveness of remediation and future research directions for the development of sustainable and effective pollutant cleaning strategies (Alloway, 2013a). Whereas several recent reviews have explored different facets of microbial remediation, like the role of certain pollutants and bioremediation techniques, the current review offers a holistic view by considering not only various pollutants but also the mechanism of microbes, geo-environmental engineering, monitoring techniques, and advanced technology. The current scope and interdisciplinary approach make the current review unique among previous publications on microbial remediation and point toward future directions for sustainable soil remediation. The literature search was performed in Scopus, Web of Science, ScienceDirect, PubMed, and Google Scholar. Publications from 2000 to 2025 were considered, with older studies retained only when they established mechanisms that remain standard in the field, such as classical biodegradation pathways, reductive dehalogenation, biosorption, or biomineralization. The same core search structure was applied across databases, with minor syntax changes required by individual database interfaces: (“microbial remediation” OR “bioremediation”) AND (“recalcitrant pollutants” OR “persistent pollutants” OR “soil contamination”) AND (“PAHs” OR “petroleum hydrocarbons” OR “heavy metals” OR “metalloids” OR “PFAS” OR “pesticides” OR “microbial consortia” OR “geo-environmental engineering”). Additional pollutant-specific searches were performed for “microbially induced carbonate precipitation,” “bioelectrochemical remediation,” “microplastic soil remediation,” “PFAS biotransformation,” “omics monitoring,” and “synthetic biology bioremediation.” Records were screened first by title and abstract, and then by full text when the article addressed microbial processes, pollutant transformation or immobilization mechanisms, soil or geoenvironmental matrices, laboratory or field-scale remediation, monitoring endpoints, or emerging microbial technologies. Publications were excluded when they focused only on non-microbial treatment, did not address soil, sediment, groundwater, or geoenvironmental systems, or lacked sufficient methodological detail to support the claim for which they would be cited. This article is a narrative review rather than a systematic review. Therefore, record counts and PRISMA-style screening statistics were not used.

## 2 Recalcitrant pollutants in soil and geo-environmental systems

### 2.1 Definition and characteristics of recalcitrant pollutants

Recalcitrant pollutants are environmental contaminants that do not break down easily through physical, chemical, and biological processes. Because of their stable molecules, low availability, and relatively small ability to be degraded by microbes, these pollutants remain in soil and geo-environmental systems for extended periods. Recalcitrant pollutants accumulate in the environment and can be transported far from their source, causing significant ecological and health impacts (Megharaj et al., 2011), while easily degradable compounds do not. Their persistence is largely due to their physical and chemical characteristics, including hydrophobicity, molecular complexity, presence of halogens, high molecular weight, and low water solubility. These prevent natural decay and access by microorganisms. Recalcitrant compounds often have a strong affinity for soil particles and organic matter and thus are less readily degraded. Another important characteristic of recalcitrant pollutants is that they are toxic to soil organisms and other living things. As a consequence of long-term exposure, microbial community structures may change, biochemical processes of the soil may be disrupted, and nutrient cycling may be changed (Schwarzenbach et al., 2010). A large number of persistent pollutants are also known to cause cancers, genetic mutations, birth defects, and disrupt the endocrine system. These pollutants are thus an important issue for environmental remediation and sustainable land use.

### 2.2 Sources of recalcitrant pollutants

Recalcitrant pollutants can result from a variety of human actions such as manufacturing in industries, oil refining, mining, agriculture, production of medications, discharge of wastewater, effluent from landfills, and poor disposal of wastes (Richardson and Ternes, 2018). Oil spills, pesticides and fertilizers used, burning of fossil fuels, and emission of untreated effluents from industries also play a major role in causing pollution of soil and geo-environment (Ling et al., 2026), (Gavrilescu, 2005).

### 2.3 Classification of recalcitrant pollutants

**FIGURE 2:**
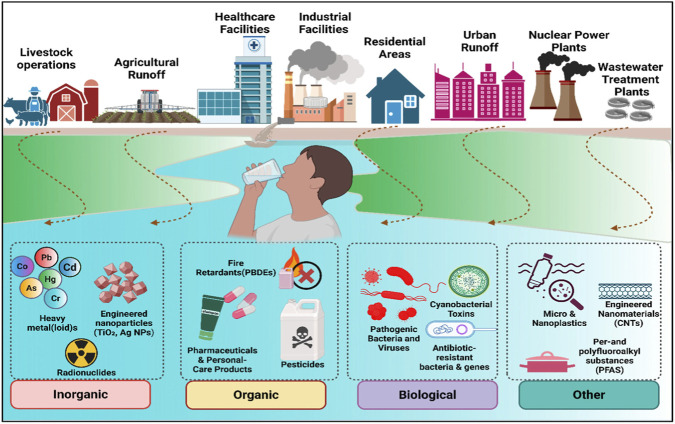
Broad source-oriented overview of environmental contaminants affecting soil and water systems. The working classification used in this review remains organic pollutants, inorganic pollutants, and polymeric or particulate contaminants; emerging pollutants are treated as a cross-cutting descriptor rather than as a chemical class (Singh et al., 2025).

The soil and geo-environmental systems consist of three types of contaminants which are very resistant to degradation: (i) organic pollutants, (ii) inorganic pollutants, and (iii) polymeric or particulate contaminants. Organic pollutants are comprised of polycyclic aromatic hydrocarbons (PAH), petroleum hydrocarbons, pesticides, chlorinated compounds, pharmaceuticals, and per- and polyfluoroalkyl substances (PFAS). Heavy metals/metalloids are inorganic pollutants. Microplastics and nanoplastics are the major polymeric or particulate contaminants. Emerging pollutants are not a chemical class, but rather one of the cross-cutting terms used in this review to describe contaminants increasing in environmental concern and regulatory interest. Figure 2 is used only as a broad source-oriented overview of environmental contaminants that may enter soil and water systems. It is not intended to define the chemical classification adopted in this review. In the present review, recalcitrant pollutants are discussed under three working groups: organic pollutants, inorganic pollutants, and polymeric or particulate contaminants. Emerging pollutants may occur across these groups and are treated here as a cross-cutting label based on environmental occurrence, persistence, risk concern, and regulatory attention.

#### 2.3.1 Organic pollutants

Recalcitrant organic pollutants comprise one of the largest categories of pollutants owing to their complex chemical structure and difficult biodegradation processes. Such pollutants may result from various industrial, agricultural, and petroleum-based activities. Among the most studied pollutants are the polycyclic aromatic hydrocarbons (PAHs) (Safe, 1994). The formation of such hydrocarbons occurs due to incomplete combustion of fossil fuels, biomass, and other organic substances. The hydrophobicity of the compounds and their carcinogenic activity make PAHs among the highest priorities as regards environmental pollution. Petroleum hydrocarbons usually enter water systems *via* spills of oil and fuel, refining, and transportation processes (Sharma, 2020). Pollution by such compounds poses a threat to soil quality and underground water. Another pollutant group includes polychlorinated biphenyls, chlorinated solvents, and organochlorine pesticides. Their high level of stability and persistence is coupled with their ability to bioaccumulate and biomagnify along the food chain. Synthetic dyes produced during operations of various industries, including textile, leather, paper, and dye production, are among organic pollutants (Rillig, 2012). The majority of the dyes possess an aromatic structure and are resistant to degradation. Pharmaceuticals and PFAS are also treated as organic contaminants because of their carbon-based structures and environmental behavior. They are discussed additionally as emerging contaminants because of their increasing environmental detection, persistence, biological effects, and regulatory concern. Microplastics and nanoplastics are not classified here as organic pollutants in the same sense as PAHs, pesticides, pharmaceuticals, or PFAS. They are treated as polymeric or particulate contaminants, while also being discussed within the broader group of contaminants of emerging environmental concern. In this review, “emerging pollutants” is therefore used as a cross-cutting environmental descriptor, not as a chemical class.

#### 2.3.2 Emerging contaminants of environmental concern

**FIGURE 3:**
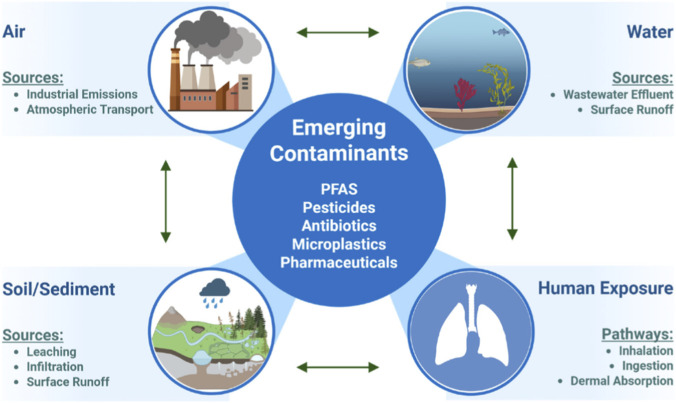
Major categories and environmental sources of emerging pollutants, including pharmaceuticals, personal care products (PPCPs), PFAS, microplastics, and nanoplastics (Sujan Sai et al., 2026).

Emerging pollutants (EPPs) are contaminants that have drawn more scientific interest because of their widespread presence, persistence, and potential effects on the environment and human health. Unlike traditional pollutants, many EPPs are not fully regulated, even though there is increasing evidence of their risks to the environment. A major subgroup includes pharmaceuticals and personal care products (PPCPs). These enter soils through wastewater irrigation, biosolid application, and landfill leachate (Chandel et al., 2025). Commonly detected PPCPs include antibiotics, pain relievers, hormones, and antiseptics. Another significant class includes per- and polyfluoroalkyl substances (PFAS), such as perfluorooctanoic acid (PFOA) and perfluorooctane sulfonic acid (PFOS). These are often called “forever chemicals” due to their strong chemical stability and resistance to breakdown. Additionally, microplastics and nanoplastics have become important contaminants (Rillig, 2012). They can carry harmful substances and disrupt soil properties, microbial communities, nutrient cycling, and pollutant behavior. Figure 3 illustrates the major categories of emerging pollutants and their common environmental sources. These contaminants are increasingly detected in soil and groundwater and have attracted attention because of their persistence and potential ecological risks.

#### 2.3.3 Heavy metals and metalloids

Heavy metals and metalloids are classified as inorganic pollutants that cannot be broken down. Instead, microbial remediation aims to change their forms, movement, availability to living organisms, and harmfulness through processes such as biosorption, bioaccumulation, oxidation and reduction reactions, immobilization, and biomineralization. Remediation techniques are aimed at their immobilization, transformation, or removal. Some of the common heavy metals include cadmium, lead, chromium, mercury, copper, nickel, and zinc, and some of the common metalloids include arsenic and selenium (Tchounwou et al., 2012). Major sources include mining, industrial emissions, contaminated effluents, fertilizer application, and poor waste management. Heavy metals alter microbial community composition, inhibit enzymatic reactions, reduce soil fertility, and have negative impacts on plant development. When these pollutants build up in crop species, human diseases ranging from neurodegenerative conditions, kidney problems, and cancer emerge (Alloway, 2013b). Microbial remediation of heavy metals and metalloids proceeds through distinct processes, including biosorption, bioaccumulation, biomineralization, oxidation, reduction, methylation, and immobilization. Biosorption and immobilization can lower risk when they decrease dissolved or exchangeable contaminant fractions and reduce mobility and bioavailability. Bioaccumulation may reduce exposure when contaminants are retained within microbial biomass, although biomass stability and contaminant release after cell death must be considered. Biomineralization can stabilize metals and metalloids through insoluble carbonate, phosphate, sulfide, or oxide phases, but its permanence depends on pH, redox potential, competing ligands, and mineral stability. Oxidation, reduction, and methylation reactions can either decrease or increase risk depending on the resulting species. Microbial methylation of mercury can produce methylmercury, a more toxic and bioaccumulative form, while microbial transformations of arsenic can alter mobility and toxicity under different geochemical conditions. Remediation outcomes should therefore be evaluated from speciation, mobility, bioavailability, persistence, toxicity, and overall environmental risk, not from transformation alone.

## 3 Soil microbial communities involved in remediation

**FIGURE 4:**
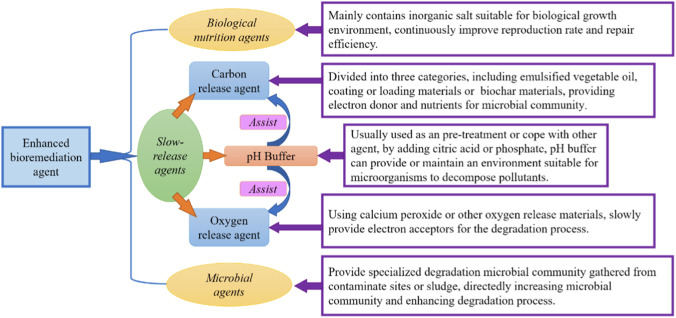
Categories and main objectives of enhanced *in situ* bioremediation agents for organic pollution remediation in groundwater (Xie et al., 2024).

**FIGURE 5:**
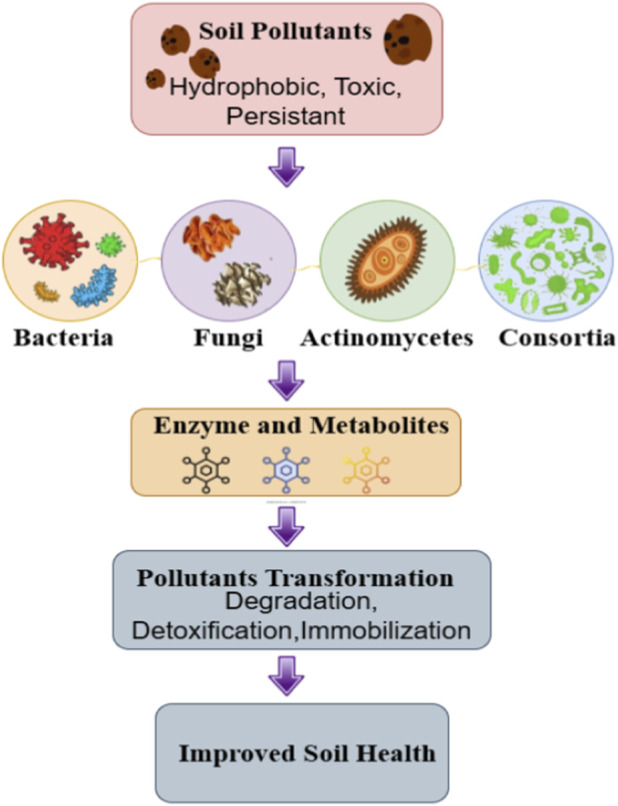
Conceptual overview of the major soil microbial groups involved in pollutant degradation and remediation.

Soils are biologically degraded by microorganisms, who play an important role in the detoxification, degradation, and transformation of contaminants in soils. The high biodiversity of microorganisms within soils is useful for performing vital functions in ecosystems including the degradation and mineralization of organic matter, nutrient cycling, and bioremediation of soils. Thanks to their versatile metabolism, soil microorganisms have the potential to make use of contaminants as a source of carbon and energy or to alter contaminants to render them less toxic. Bacteria, fungi, actinomycetes, and microbial consortia are key groups of microbes that break down tough contaminants. To boost the effectiveness of these organisms in the field, enhanced *in situ* bioremediation strategies use different amendments and support agents that increase microbial activity and speed up contaminant breakdown. The main types of these EISB agents for groundwater and soil cleanup are shown in Figure 4. Figure 5 provides a conceptual overview of the major microbial groups involved in pollutant degradation and their complementary roles in microbial remediation.

### 3.1 Bacteria

**FIGURE 6:**
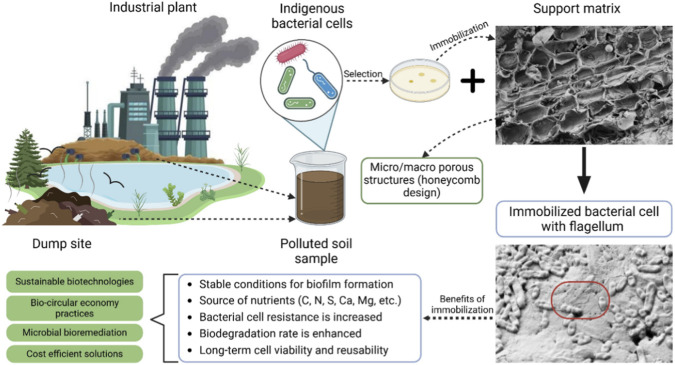
Representative bacterial mechanisms involved in microbial remediation of soil contaminants, including biodegradation, biosorption, bioaccumulation, and enzymatic transformation (Sujan Sai et al., 2026).

Bacteria are among the most studied microorganisms in bioremediation because many taxa grow rapidly, express diverse catabolic enzymes, and adapt to contaminant exposure. Some bacterial species express enzymes that degrade complex organic contaminants (Das and Chandran, 2011). Examples of genera that are known for their degradation activities include *Pseudomonas, Bacillus, Rhodococcus, Acinetobacter, Sphingomonas, and Burkholderia.* Bacteria use contaminants as sources of carbon and energy for both aerobic and anaerobic degradation processes (Gadd, 2004). In addition to decomposition activities, some microorganisms are capable of transforming and even immobilizing metals using the oxidation-reduction reaction, biosorption, precipitation, and bioaccumulation processes (Poblete-Castro et al., 2012). The principal bacterial pathways involved in contaminant removal are summarized in Figure 6. These mechanisms enable bacteria to degrade or transform a wide variety of organic pollutants and immobilize certain inorganic contaminants.

### 3.2 Fungi

Fungi have long, branching hyphal structures that can reach contaminants in deep soils that would not otherwise be accessible to bacteria. This allows increased exposure and enhanced efficiency of pollutant degradation. Most fungi are capable of secreting extracellular enzymes with wide substrate specificity, like lignin peroxidase, manganese peroxidase, laccase, and versatile peroxidase (Das and Chandran, 2011). These enzymes are effective at degrading complex and recalcitrant chemicals that are often difficult for bacteria to degrade (Viswanath et al., 2014). Among the well-known fungi that degrade various pollutants are white-rot fungi like those belonging to the genera Phanerochaete, Trametes, and Pleurotus. In addition to degrading various pollutants, fungi can help remove heavy metals from soils using biosorption, bioaccumulation, and immobilization processes (Pointing, 2001).

### 3.3 Actinomycetes

Actinomycetes are a diverse group of filamentous bacteria. Actinomycetes are widely distributed in soil environments and contribute significantly towards decomposition processes in soil. Species within genera like *Streptomyces*, *Nocardia*, and Micromonospora possess great metabolic capability, allowing them to degrade resistant organic materials (Arenskötter et al., 2004). They are capable of producing various extracellular enzymes that aid in the degradation of hydrocarbons, pesticides, chlorinated compounds, and other pollutants. Actinomycetes contribute significantly to the biodegradation of hydrophobic substances, partly through the production of biosurfactants and other extracellular metabolites that enhance pollutant bioavailability. Their stress resistance also makes Actinomycetes suitable for prolonged remediation efforts in contaminated soils. The increasing focus on using actinomycetes in environmental decontamination is due to their wide array of enzymes and tolerance to polluted environments (Goodfellow and Williams, 1983). Furthermore, only a small part of the metabolic diversity and genetic makeup of actinomycetes has been fully studied. Current genomic and metagenomic research indicates that many unannotated genes might code for new enzymes that help break down tough organic pollutants. This shows the significant potential of actinomycetes for bioremediation in the future.

### 3.4 Microbial consortia

Degradation of pollutants, in natural settings, rarely occurs with the involvement of a single microorganism, but involves communities and consortia of microbes where the breakdown of pollutants takes place due to a combination of metabolisms of different types of microbes. Consortia of microbes involve multiple species that cooperate in degrading the pollutants effectively compared to single strains (Tyagi et al., 2011). Pollutants can be broken down step by step through the cooperation of different microbes in order to break down the complex pollutants into smaller molecules, which can further be utilized by another member of the community. The use of microbial consortia can bring several advantages like wide substrate utilization, improved degradation, improved adaptability, and greater resistance to toxic stress (Xu et al., 2018). Mixed culture microbial populations perform better than single strains for petroleum hydrocarbons, PAHs, pesticides, dyes, pharmaceuticals, and contaminated systems.

**FIGURE 7:**
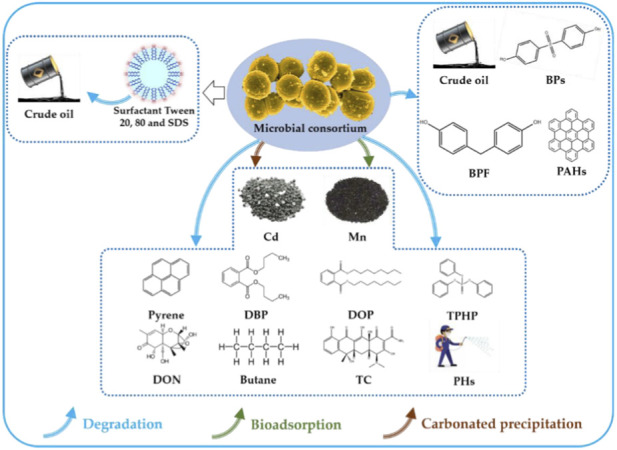
Synergistic interactions among microbial consortia during sequential degradation of recalcitrant pollutants in contaminated soils (Sujan Sai et al., 2026).

A recent study indicated that the microbial consortia generally perform better than the single strains due to the metabolic interactions they have and the wider substrates they can utilize. But it is still challenging to achieve the stable composition of the consortium under field conditions, which requires further optimization for large-scale application. As shown in Figure 7, microbial consortia use complementary metabolic pathways and cross-feeding interactions that enhance the degradation efficiency of complex pollutants compared with single microbial strains.

Published studies support the mechanistic advantage of microbial consortia over single strains, but this advantage depends on contaminant chemistry, environmental conditions, and community stability. In PAH-contaminated systems, consortia containing genera such as *Pseudomonas*, Rhodococcus, Sphingomonas, and *Mycobacterium* may combine ring-hydroxylating dioxygenases, dehydrogenases, catechol dioxygenases, and downstream central metabolic pathways. One member may initiate oxidation of phenanthrene, pyrene, or related PAHs to dihydrodiols, while other members transform intermediates such as catechol, protocatechuate, or phthalate through ortho- or meta-cleavage reactions before entry into central carbon metabolism. Such syntrophic division of labor can reduce bottlenecks caused by substrate specificity, metabolite toxicity, or incomplete pathway expression in single isolates. In petroleum-contaminated soils, hydrocarbon-degrading bacteria can act with biosurfactant-producing populations that increase contaminant bioavailability, while aerobic conditions, moisture, and nitrogen and phosphorus availability control the rate and extent of removal (Das and Chandran, 2011). Similar consortium logic applies to organophosphate pesticide degradation, where initial hydrolysis products may be further metabolized by other community members. These examples show that consortium performance should be reported with the pollutant identity, starting concentration, matrix, incubation period, microbial partners, measured intermediates, analytical endpoint, and control treatment. Without those details, percentage removal values should be interpreted cautiously, especially when studies measure only parent compound loss rather than.

### 3.5 Adaptation of microorganisms to polluted environments

Microorganisms inhabiting contaminated environments are exposed to toxic compounds, nutrient limitation, redox variation, osmotic stress, and other physicochemical pressures. These conditions can select for populations that tolerate, transform, immobilize, or metabolize specific contaminants. Adaptation at the community level may involve mutation, enrichment of tolerant taxa, horizontal gene transfer, activation of stress-response systems, altered membrane composition, and induction of catabolic enzymes. Plasmids, transposons, and other mobile genetic elements can transfer genes encoding oxygenases, dehalogenases, hydrolases, reductases, efflux systems, metal-resistance determinants, and stress-protection proteins between microbial populations (Flemming and Wingender, 2010). Biofilm formation further supports survival by protecting cells from toxic exposure, retaining nutrients, concentrating extracellular enzymes, and facilitating metabolite exchange among community members. During prolonged contaminant exposure, microorganisms with relevant catabolic or resistance traits may increase in relative abundance, causing shifts in microbial community composition and the emergence of specialized pollutant-transforming assemblages (Top and Springael, 2003). These processes support natural attenuation and engineered bioremediation, but they do not guarantee sustained field performance. Environmental variability, competition with indigenous communities, contaminant bioavailability, and nutrient or electron acceptor limitation can still restrict remediation outcomes (Flemming et al., 2016).

## 4 Mechanisms of microbial remediation

Microbial remediation includes degradation, transformation, immobilization, and detoxification processes. Degradation refers to the enzymatic breakdown of complex organic contaminants into simpler products and, when complete, to mineralization to CO_2_, H_2_O, inorganic ions, and microbial biomass. Transformation produces chemically different compounds or elemental species; it may lower risk, but it may also increase mobility, persistence, or toxicity depending on the products formed. Immobilization decreases contaminant mobility or bioavailability through sorption, precipitation, complexation, or mineral entrapment. Detoxification should be used only when a decrease in hazard, toxicity, or biological effect has been demonstrated. Transformation alone is therefore not evidence of detoxification. Microbial remediation may proceed through biosorption, bioaccumulation, enzymatic transformation, biomineralization, co-metabolism, syntrophic metabolism, or biofilm-mediated processes. Laboratory studies with hydrocarbon-degrading bacteria and metal-binding fungal biomass demonstrate these mechanisms under controlled conditions, whereas field and *in situ* applications require additional evidence from contaminant mass balance, transformation products, residual toxicity, geochemical stability, and microbial functional activity.

### 4.1 Biodegradation

Microbial remediation *via* biodegradation is one of the best-studied microbial processes (Bala et al., 2022). It includes a transformation of complex organic contaminants into simpler, non-toxic products by means of microbial metabolism. The pollutants serve as substrates of carbon, energy, and other nutrients for microorganisms. Thus, biodegradation occurs gradually. It can be carried out in both aerobic and anaerobic environments (Das and Chandran, 2011). In aerobic biodegradation, microorganisms take oxygen from the atmosphere to oxidize the pollutants to carbon dioxide and water along with microbial growth. Aerobic biodegradation is very effective against petroleum hydrocarbons, PAHs, and some pesticides. Anaerobic biodegradation occurs when microorganisms use various substitutes to replace molecular oxygen as the terminal electron acceptor, such as nitrate, sulfate, ferric iron, or carbon dioxide. Anaerobic biodegradation is highly efficient at decomposing chlorinated compounds as well as pollutants in low oxygen concentrations. Effective biodegradation relies upon microorganism diversity, availability of pollutants, environmental conditions, and nutrients (Singh and Ward, 2004). Because it is environmentally friendly and can fully mineralize contaminants, biodegradation remains a foundation of bioremediation technologies. The biodegradation process starts with the attack of complex pollutant molecules by extracellular or intracellular enzymes, which generate intermediate metabolites that are then converted into central metabolic pathways, e.g., the tricarboxylic acid (TCA) cycle. The final mineralization stage will release and destroy contaminants into CO_2_, water, inorganic ions, and microbial biomass, which will decrease their environmental toxicity.

Microbiological degradation has been proven to be a good method for treating hydrocarbon polluted soils in recent works. *Pseudomonas* and Rhodococcus spp. are reported to be efficient degraders of petroleum hydrocarbons and PAHs *via* oxygenase pathways, making them promising for sustainable bioremediation in the field. Biodegradation is very effective for many organic contaminants, but varies depending on contaminant structure and environmental conditions. Recent research shows that high molecular weight PAHs and fluorinated compounds are difficult to mineralize and can only be partially degraded, which further emphasizes the need for engineered microbial systems and combined remediation strategies.

### 4.2 Biosorption

Biosorption refers to the attachment of contaminants to the surface of microorganisms using physical and chemical processes. While biodegradation involves active metabolism, biosorption neither needs active metabolism nor living microorganisms, as living and dead microbes are equally effective in this case. There are different types of functional groups present in the cell walls of microbes, such as carboxyl, hydroxyl, amino, phosphate, and sulfhydryl groups that are responsible for attaching contaminants to cell surfaces (Wang and Chen, 2009). Using ion exchange, chelation, electrostatic forces, and adsorption, microorganisms can efficiently clean up contaminated environments by eliminating heavy metals as well as certain organic contaminants. Some examples of toxic metals that could be effectively removed using the biosorption approach include lead, cadmium, chromium, copper, mercury, and arsenic (Hlihor et al., 2022). Various factors like pH, temperature, biomass concentration, contact time, and physicochemical properties of the contaminant affect the efficiency of biosorption. Biosorption is a relatively inexpensive and quick method for removal of metals in metal-contaminated environments as it does not depend on metabolically active cells.

### 4.3 Bioaccumulation

In bioaccumulation, there is active accumulation and storage of pollutants in microorganisms. This is different from biosorption since in bioaccumulation, there must be living microorganisms with the ability to metabolize. The microorganism takes up the pollutants from their environment and stores them in the cells. Metals can be stored in vacuoles, proteins, or reduced to non-toxic forms. Metal-binding agents such as metallothioneins and phytochelatins have been produced by microorganisms in order to aid in the bioaccumulation process. Bioaccumulation assists in removing toxic metals and metalloids in polluted soils and waters. However, the efficiency of the process relies on microbial viability among others (Bruins et al., 2000).

Bioaccumulation is an energy-dependent microbial metabolic process, whereas passive biosorption is not. The storage of contaminants in the cell lowers the contaminants’ bioavailability and helps to detoxify polluted environments over the long-term.

### 4.4 Biotransformation

Biotransformation is an activity whereby enzymes work by transforming contaminants into products that are chemically modified, which are often less toxic, immobile, and more susceptible to further degradation. In biotransformation, the reactions involved do not lead to the complete degradation of contaminants but form intermediate compounds that are subject to further degradation by microorganisms. Biotransformation involves the use of several enzymes such as oxygenases, dehydrogenases, reductases, hydrolases, and peroxidases in transformation processes. Transformation reactions include oxidation, reduction, hydrolysis, methylation, dehalogenation, and dealkylation. Biotransformation plays an essential role in the clean-up of persistent organic pollutants such as pesticides, pharmaceutical products, dyes, and chlorinated solvents. Mostly, these transformations tend to make contaminants less toxic and biodegradable (Van der Oost et al., 2003). Biotransformation is often preceded by complete biodegradation, but in many cases occurs as an intermediate step, which results in the creation of compounds more prone to microbial degradation. This mechanism is important for degradation of complex organic pollutants in terms of their toxicity and persistence.

### 4.5 Biomineralization

Biomineralization is defined as the biological mechanism in which microorganisms aid in the formation of mineral phases that contain or modify contaminants. It is crucial in the decontamination of hazardous metals and radionuclides. Biochemical processes from microorganisms can affect the geochemistry of an area, resulting in the formation of carbonate, phosphate, sulfide, and oxide minerals. Such minerals are capable of immobilizing contaminants, reducing their mobility, and making them unavailable. There is a popular biomineralization process referred to as Microbially Induced Carbonate Precipitation (MICP). The process involves ureolytic bacteria generating carbonate ions that interact with calcium ions and lead to the precipitation of calcium carbonate (Achal et al., 2011). Apart from stabilizing soil, MICP is also used to entrap toxic metals within mineral phases. In addition to carbonate precipitation, microorganisms can also cause the precipitation of toxic metal-immobilising phosphate and sulfide minerals. These fixed mineral phases limit the movement of contaminants and help to prevent prolonged groundwater contamination. Ureolytic bacteria, including Sporosarcina pasteurii, have been recently found to precipitate calcium carbonate and to efficiently immobilize heavy metals, decreasing their mobility and environmental risk. The results further confirm the use of MICP as a potential technology for geo-environmental remediation.

### 4.6 Co-metabolism

The transformation of contaminants in the course of decomposition of a primary substrate by microorganisms is referred to as co-metabolism. In this context, contaminants themselves do not serve as an energy source, but are degraded as a result of the action of enzymes involved in the metabolism of some other compound. In many cases, contaminants are not usable for the purpose of microorganism growth on their own (Pandolfo et al., 2023). If there are proper co-substrates such as methane, toluene, glucose, or phenol, then the organisms release enzymes capable of destroying these contaminants. The examples of substrates for co-metabolism include chlorinated solvents, pesticides, pharmaceuticals, and aromatic hydrocarbons (Semprini, 1997). Despite its effectiveness, co-metabolic degradation often requires careful management of nutrients and environmental conditions to keep microorganisms active. Co-metabolic degradation is a process that is largely dependent on the availability of appropriate growth substrates and enzyme induction. This mechanism is especially useful for contaminants that do not themselves establish a nutritional source for microbial growth but can be converted as a by-product of the metabolism of another nutrient.

### 4.7 Biofilm-mediated remediation

Biofilms are communities of microbes that settle on a surface and are protected by a polymer matrix that they secrete themselves. It is often seen as a survival mechanism by microorganisms in polluted environments. Biofilms can improve remediation performance under some conditions. The matrix protects the microbes that live in the biofilm from predators and pollutants. The biofilm also provides a habitat for many microorganisms, which allows for cooperation to metabolically remove the pollutant that is causing the contamination. The biofilm will absorb pollutants and bring them closer to the microorganisms, therefore improving the removal of the pollutant (Singh et al., 2006). Biofilms also allow for the transfer of genes, which can result in more metabolic capabilities for degradation. Biofilms have proven to be effective when trying to remove pollutants of hydrocarbons, heavy metals, PAHs, dyes, and other contaminants. Biofilm based processes are increasingly applied in bioreactors, permeable reactive barriers, and other engineered remediation systems (Flemming et al., 2016). The extracellular polymeric substances (EPS) of biofilms facilitate microbial adhesion, bind nutrients, and shield cells from environmental stress and toxic chemicals. Biofilm systems also usually achieve higher pollutant removal efficiencies and are more stable than free-living microbial populations.

## 5 Microbial remediation of major recalcitrant pollutants

Microbial remediation has been widely studied for recalcitrant pollutants in contaminated soils and geoenvironmental systems. The effectiveness of any microbial process largely depends upon the properties of pollutants, availability of diverse species of microorganisms, environmental factors, and presence of metabolic processes. Various kinds of microorganisms possess different capabilities for the remediation of pollutants, enabling the biodegradation of a wide range of organic contaminants and the transformation, immobilization, or detoxification of inorganic pollutants. This section examines microbial remediation of major recalcitrant pollutants and separates degradation, transformation, immobilization, and detoxification endpoints. Recent studies have reported successful microbial remediation of several recalcitrant pollutants using bacterial and fungal species. For instance, *Pseudomonas, Rhodococcus, and Sphingomonas* have been widely investigated for PAH degradation, while microbial consortia have demonstrated improved removal efficiencies through synergistic metabolic activities. These published findings highlight the significant potential of microbial technologies for environmental cleanup.

### 5.1 Polycyclic aromatic hydrocarbons (PAHs)

**FIGURE 8:**
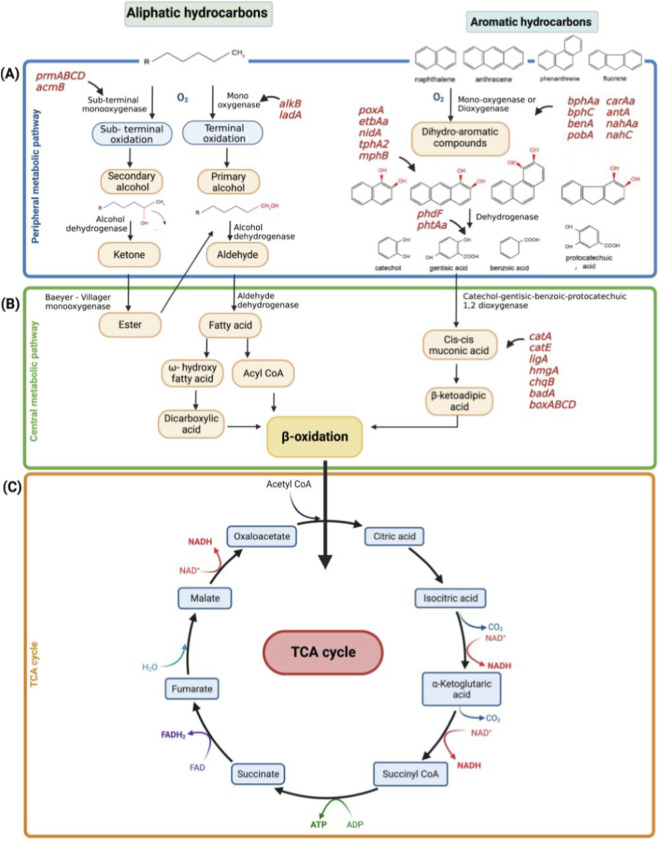
Metabolic pathway diagram showing the degradation of aliphatic and aromatic hydrocarbons. **(A)** illustrates peripheral metabolic pathways for aliphatic and aromatic hydrocarbon oxidation, identifying key enzymes and intermediates, with gene names in red. **(B)** details central metabolic pathways through β-oxidation, showing intermediates like esters, fatty acids, dicarboxylic acid, and cis-cis muconic acid, and referencing key enzymes. **(C)** portrays the tricarboxylic acid (TCA) cycle, indicating conversion steps, involved molecules such as ATP and NADH, and essential intermediates including oxaloacetate, citric acid, and succinate (Pandolfo et al., 2023).

PAHs refer to environmentally persistent organic pollutants that consist of two or more fused aromatic rings. The formation of PAHs occurs mainly from the incomplete combustion of fossil fuels, petroleum products, biomass, and industrial activities (Cerniglia, 1993). Owing to their hydrophobicity, poor aqueous solubility, and high chemical stability, the accumulation of PAHs in soils is likely to pose great environmental and health threats. Microbial transformation is considered one of the most efficient methods to degrade PAHs. Several bacterial genera such as *Pseudomonas*, Sphingomonas, *Mycobacterium*, Rhodococcus, and *Bacillus* are capable of transforming PAHs using oxidative enzymes. The initial steps in PAH degradation involve ring-hydroxylating dioxygenases, which add oxygen to the aromatic ring to produce cis-dihydrodiols. Subsequent dehydrogenation and ring cleavage occur before catabolites are metabolized through central metabolic pathways. Fungi, especially white-rot fungi such as Phanerochaete chrysosporium and *Trametes versicolor*, have also been found to be effective due to their extracellular oxidative enzymes (Kanaly and Harayama, 2000). The efficiency of such organisms has been shown experimentally in the process of PAH bioremediation. For instance, such species as *Pseudomonas* spp. and *Mycobacterium* spp. are known for their capability to degrade PAH molecules of lower weight in aerobic conditions. High molecular-weight PAH degradation can be facilitated by microbial consortia due to sequential metabolization (Sakshi and Haritash, 2020). Several laboratory and field experiments have clearly shown effective removal of PAHs by microorganisms, provided suitable conditions prevail; however, degradation efficacy is affected by contaminant composition and environment. In general, microbial transformation consists of the initial oxygenation of PAH structures, followed by successive dehydrogenations and mineralizations. While PAHs with lower molecular weights tend to be easily biodegraded, those with higher molecular weights require special microbial populations and extended cleanup periods (Haritash and Kaushik, 2009). Similar processes are used for the breakdown of other hydrocarbon compounds by microbes, except that different enzyme reactions and microbial types may be used, depending upon the structural nature of the pollutant. In published studies, the low molecular weight PAHs have been observed to be more easily degraded than high molecular weight PAH. That indicates that, when dealing with more persistent PAH compounds, a microbial consortium or integrated remediation technology might be required for effective removal. Under laboratory conditions, bacterial isolates and mixed consortia can reduce concentrations of low-molecular-weight PAHs more readily than high-molecular-weight PAHs, but reported efficiencies vary with PAH concentration, soil organic matter, aging of contamination, moisture, oxygen availability, nutrient status, incubation time, and analytical endpoint. Studies with *Pseudomonas*, *Mycobacterium*, Rhodococcus, Sphingomonas, and mixed consortia show that naphthalene and phenanthrene generally degrade faster than pyrene, chrysene, benzo [a]pyrene, and other high-molecular-weight PAHs because sorption strength and low aqueous solubility reduce bioavailability. The mechanistic sequence usually begins with ring-hydroxylating dioxygenases or fungal oxidative enzymes, followed by dihydrodiol formation, dehydrogenation, ring cleavage, and conversion of intermediates such as catechol, protocatechuate, phthalate, salicylate, or gentisate into central metabolic pathways. However, parent compound loss does not by itself prove mineralization or detoxification. PAH remediation should be assessed by separate endpoints: decrease in parent PAH concentration, formation and persistence of metabolites, mineralization to CO_2_ and biomass where measured, residual toxicity, and change in environmental risk. GC-MS, HPLC, or GC-FID can quantify parent PAHs and selected metabolites, but mineralization requires additional evidence, such as CO_2_ evolution, isotope tracing, or mass balance. If the cited studies measured only parent compound reduction, the manuscript should describe the outcome as removal or transformation rather than complete degradation or detoxification. Figure 8 shows, the microbial biodegradation pathway of polycyclic aromatic hydrocarbons (PAHs) through enzymatic oxidation, ring cleavage, and mineralization (Pandolfo et al., 2023).

### 5.2 Petroleum hydrocarbons

**FIGURE 9:**
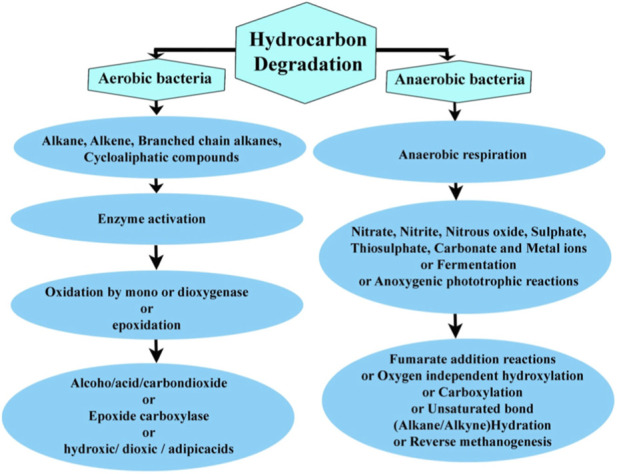
Microbial degradation pathways of petroleum hydrocarbons mediated by hydrocarbon-degrading bacteria and fungi (Radhakrishnan et al., 2023).

Hydrocarbons derived from petroleum are some of the most frequent contaminants found in soils due to oil spills, fuel leaks, petroleum refining, transport incidents, and various industrial operations. These compounds comprise different chemicals such as alkanes, cycloalkanes, aromatic hydrocarbons, and asphaltenes (Das and Chandran, 2011). There are various kinds of microorganisms able to degrade petroleum hydrocarbons by utilizing these as their source of energy and carbon. Some of the hydrocarbon-degrading microorganisms include species such as *Pseudomonas*, Alcanivorax, *Acinetobacter*, *Bacillus*, and Rhodococcus. The microorganisms involved in hydrocarbon decomposition have enzymes such as mono-oxygenases and di-oxygenases, which initiate the process of oxidation of the hydrocarbons (Varjani, 2017). Aliphatic hydrocarbons are metabolized through the action of alkane hydroxylases, while aromatic hydrocarbons are subjected to oxidation by the action of mono-oxygenases and di-oxygenases followed by β-oxidation. Biosurfactant-producing microbes contribute to the degradation of hydrocarbons by making these more soluble and available for degradation. Consortia of microorganisms have been employed in biodegradation owing to the ability to metabolically complement each other, thus improving the decomposition of hydrocarbons (Prince, 2015). Comparative soil studies show that petroleum hydrocarbon bioremediation is strongly site-specific. In a controlled incubation study of field-contaminated diesel soils collected from Long Beach, California, and Hong Kong, Bento et al. compared natural attenuation, nutrient-based biostimulation, and bioaugmentation for 12 weeks. Total petroleum hydrocarbons were separated into light C12-C23 and heavy C23-C40 fractions, and microbial activity was followed with a dehydrogenase assay. In the Long Beach soil, bioaugmentation gave the highest degradation, with 72.7% removal of the light fraction and 75.2% removal of the heavy fraction. In the Hong Kong soil, natural attenuation outperformed biostimulation. These results show that inoculation or nutrient addition cannot be assumed to improve remediation at every site. Matrix properties, indigenous microbial populations, contaminant fraction, nutrient status, and treatment design should be evaluated before bioaugmentation or biostimulation is selected (Bento et al., 2005). Figure 9 summarizes the general microbial oxidation of petroleum hydrocarbons, but the performance of these pathways under field conditions depends on contaminant composition, oxygen transfer, moisture, nutrient balance, bioavailability, and microbial community structure (Radhakrishnan et al., 2023).

### 5.3 Chlorinated organic compounds

Chlorinated organics are among the most recalcitrant types of pollution that include chlorinated solvents, chlorophenols, polychlorinated biphenyls (PCBs), and other halo-aromatic compounds (Mohn and Tiedje, 1992). They were widely used in manufacturing industries, metal degreasing, electrical devices, and pesticides. Being stable and resistant to degradation makes these chemicals even harder to eliminate from the environment. Anaerobic bioremediation is based on a process of dechlorination where microbes systematically reduce the number of chlorine atoms in the molecule. Members of the genera Dehalococcoides, Desulfitobacterium, and Dehalobacter are successful dechlorinators. After this degree of degradation, aerobic microorganisms can oxidize the intermediate metabolites generated (L et al., 2013). Dechlorinated intermediate products may be further oxidized by aerobic microorganisms through oxidative pathways. Examples of anaerobic-aerobic treatments that have proven promising for remediation of chlorinated organic contaminants include the combination of anaerobic and aerobic technologies (Smidt and de Vos, 2004). Reductive dehalogenases facilitate the stepwise elimination of chlorine atoms in the absence of oxygen, forming less chlorinated molecules, which then become available for further oxidation under aerobic conditions.

### 5.4 Pesticides and herbicides

Most farming practices have resulted in the contamination of soil with pesticides and herbicides. Several pesticides possess long environmental persistence because of their stable chemical structure and are not easily degraded (Fenner et al., 2013). Soil microorganisms are key factors in the breakdown of pesticides like atrazine, glyphosate, chlorpyrifos, carbofuran, and other organochlorine pesticides. Pesticides can be degraded by bacteria like *Pseudomonas*, *Bacillus*, Arthrobacter, and Burkholderia. This is accomplished using different methods of degradation, which include enzyme hydrolysis, oxidation, reduction, and mineralization reactions. Key enzymes here are hydrolases, esterases, oxygenases, and phosphotriesterases, which act by hydrolyzing the ester/phosphoester linkages to form intermediates that will be degraded *via* microbial central metabolic pathways. Factors such as pH, temperature, and nutrient composition of the environment influence the process significantly (Singh et al., 2004; Cycoń et al., 2017). The use of microbial inoculants and engineered microbial consortia has improved pesticide remediation efficiency in agricultural soils.

### 5.5 Synthetic dyes

The presence of synthetic dyes poses a major environmental hazard since it results from the operation of industries including the textile, leather, paper, printing, and dyeing industries. Dyes contain intricate aromatic rings which are not easily biodegradable and are toxic to microorganisms, plants, and aquatic organisms (Saratale et al., 2011). Microbial degradation of dyes is accomplished using bacteria, fungi, yeast, and microbial consortium that secrete enzyme systems capable of degrading the dye structures. Enzymes like azoreductase, laccase, lignin peroxidase, and manganese peroxidase cause dye biodegradation by breaking down azo bonds or oxidizing aromatics to simpler molecules that can be mineralised. These include enzymes such as laccase, azoreductases, peroxidases, and oxygenases that help degrade chromophoric moieties and disintegrate the complex dye structure (Robinson et al., 2001). Fungi that produce white-rot have proved quite efficient due to their ability to produce a potent extracellular oxidative enzyme system. Bacteria such as *Pseudomonas*, *Bacillus*, *Aeromonas*, and *Enterobacter* have been shown to possess good potential for dye degradation (Forgacs et al., 2004).

### 5.6 Pharmaceuticals and antibiotics

Pharmaceuticals and antibiotics have been identified as environmental contaminants due to their constant discharge *via* wastewater, agricultural effluents, landfill leachates, and biosolid application (Kümmerer, 2009). The most common pharmaceutical pollutants are antibiotics, analgesics, anti-inflammatory agents, hormones, and personal care products. Their accumulation in soils could cause ecological toxicity and the development of antibiotic resistance in microorganisms (Michael et al., 2013). Biodegradation is one possible solution to this problem since different bacteria and fungi are capable of breaking down antibiotics and pharmaceutical waste products by oxidation, hydrolysis, dealkylation, and ring cleavage processes. Recent research has shown that microbial communities and advanced bioreactors may be helpful in enhancing pharmaceutical degradation (Aus der Beek et al., 2016).

### 5.7 PFAS

(PFAS) are chemically synthesized fluorinated compounds that are used in industrial applications and firefighting foams, coatings, and consumer goods. Due to the presence of the highly stable bond between carbon and fluorine, PFAS exhibit high resistance to environmental breakdown and are known as “forever chemicals”. Contamination by PFAS has become an environmental concern globally due to its recalcitrant nature (Huang and Jaffé, 2019). Remediation of PFAS using microbial processes poses significant challenges compared to other organic contaminants. Microbiological interactions associated with PFAS include biosorption or bioadsorption of PFAS by microbial biomass and the biological transformation of specific PFAS precursors into intermediary or terminal PFAS compounds (Wang et al., 2017). But there is a lack of scientific support for the concept of defluorination, in which the carbon-fluorine bonds are broken, resulting in the formation of mineralized terminal PFAS (Sunderland et al., 2019). Thus, the transformation of precursors does not always result in decreased persistence or toxicity of PFAS due to the generation of stable PFAS intermediates or terminal products (Shahid and Shafi, 2026).

### 5.8 Heavy metals and metalloids

**FIGURE 10:**
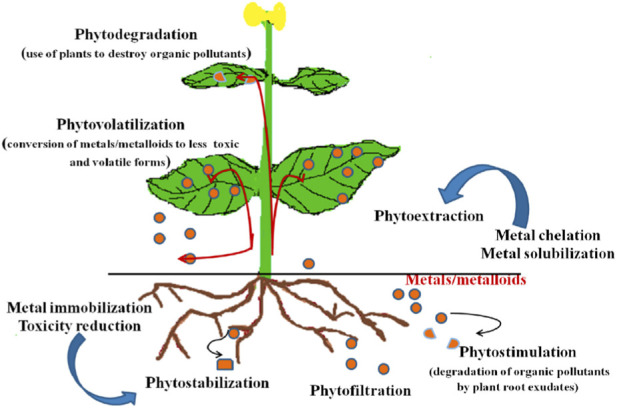
Processes used in phytoremediation of heavy metals (Ojuederie and Babalola, 2017).

Heavy metals and metalloids constitute a special class of recalcitrant pollutants as these cannot be broken down to harmless products. Rather, the approach of microbial remediation is to reduce their mobility, toxicity, and bioavailability. Typical contaminants are cadmium, chromium, lead, mercury, arsenic, nickel, copper, and zinc. There are multiple mechanisms in which microorganisms can be used for metal remediation: biosorption, bioaccumulation, bioleaching, oxidation-reduction reactions, extracellular precipitation, and others. Bacteria, like *Bacillus*, *Pseudomonas*, Shewanella, and Geobacter, are able to change the toxic form of metals into a less harmful form. Biosorption and immobilization processes also play a role through fungi and actinomycetes. Microbially induced carbonate precipitation (MICP) and sulfate-reducing bacterial systems have proven to be very promising methods for stabilization of heavy metals in contaminated soils (Dixit et al., 2015). These methods will help to decrease the mobility of contaminants and limit environmental risks, and will be used in a manner that is sustainable for cleaning up. The main mechanisms of the phytoremediation of heavy metal-polluted soils are shown in Figure 10. These processes involve phytoextraction, phytostabilization, phytovolatilization, and rhizofiltration to minimize the mobility and toxicity of metals. The effectiveness of these microbial processes depends on specific geochemical conditions at the site, including pH, redox potential, and the presence of complexing ligands. Biosorption, biomineralization, and immobilization generally reduce contaminant mobility and bioavailability. In contrast, oxidation and reduction reactions, along with changes in chemical speciation, can either decrease or increase toxicity depending on the element involved. For example, microbial methylation of mercury or transformation of arsenic species under certain conditions can improve mobility or toxicity. This highlights the need for careful evaluation of remediation outcomes (Zhang et al., 2025).

## 6 Microbial technologies in geo-environmental engineering

**FIGURE 11:**
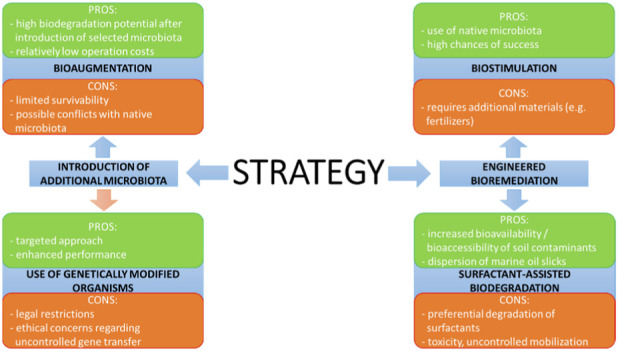
Overview of microbial technologies applied in geo-environmental engineering for sustainable soil and groundwater remediation (Ławniczak et al., 2020).

The successful remediation of contaminated soils and groundwater requires not only an understanding of microbial degradation mechanisms but also the effective application of microbial processes within engineered systems. Geo-environmental engineering integrates biological, chemical, and physical principles to develop sustainable remediation technologies capable of addressing complex environmental contamination challenges. Various microbial technologies have been developed to enhance contaminant degradation, immobilization, and stabilization in soil and subsurface environments. These approaches include natural attenuation, biostimulation, bioaugmentation, rhizoremediation, mycoremediation, microbially induced carbonate precipitation (MICP), and permeable reactive biobarriers. Each technology offers unique advantages and can be tailored to specific site conditions and contaminant characteristics. Figure 11 summarizes the major microbial remediation technologies used in geo-environmental engineering, highlighting their roles in contaminant degradation, stabilization, and ecosystem restoration.

### 6.1 Natural attenuation

**FIGURE 12:**
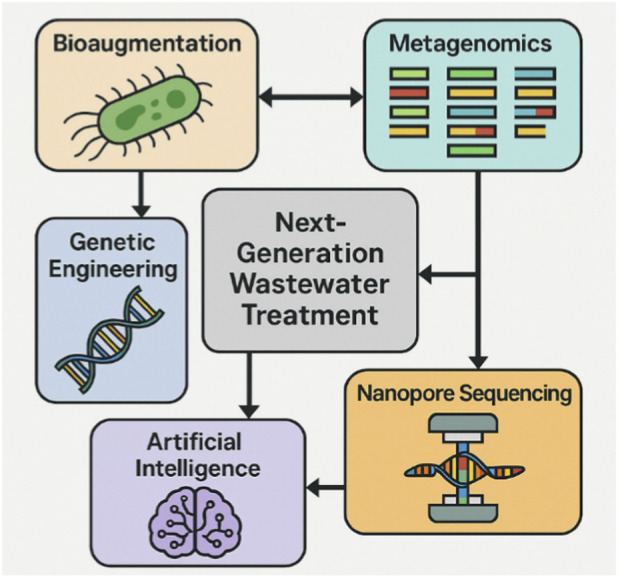
Advanced techniques used in microbial bioremediation of wastewater treatment (Mishra et al., 2025).

Natural attenuation is the process of contaminant concentration decreasing from naturally-occurring physical, chemical, and biological processes, without any direct action by humans. One of the foremost mechanisms for natural attenuation in contaminated sites is microbial degradation. Indigenous microorganisms in soil ecosystems can metabolize pollutants and gradually reduce pollutant levels over time. Dilution, dispersion, sorption, volatilization, and chemical transformation are other processes that can reduce the concentration of contaminants (Azubuike et al., 2016). Petroleum hydrocarbons, chlorinated solvents, pesticides, and some organic pollutants are known to be successfully treated *via* natural attenuation. The natural attenuation process relies on a variety of site conditions, such as microbial activity, nutrients, oxygen levels, contaminant properties, and hydrogeological conditions. This is a cost-effective and environmentally friendly process, but it is sometimes slower than engineered remediation (Wiedemeier et al., 1999). The advanced microbial technologies used in wastewater treatment and environmental remediation are shown in Figure 12. These systems augment the degradation of pollutants by biological, biochemical, and engineered microbial processes. One needs to differentiate between natural attenuation and monitored natural attenuation (MNA). While natural attenuation is characterized by the reduction of contaminants using the natural physical, chemical, and biological mechanisms available at the site without any intervention, MNA uses monitoring as part of the site management process to prove that the process results in reduction of the mass of contaminants, stabilization of the plume, degradation products, geochemical parameters, electron acceptors, and receptor exposure risk.

### 6.2 Biostimulation

Biostimulation is defined as the stimulation of existing microorganisms by introducing nutrients, electron donors, electron acceptors, and/or growth stimulators. The goal is to stimulate the populations of microorganisms that already exist and are able to break down the contaminants. However, many contaminated environments contain microorganism(s) that have the potential to remediate the pollution, but do not have the necessary nutrients or environmental conditions for effective pollutant degradation. Microbial growth and metabolic activities can be greatly stimulated by providing nitrogen, phosphorus, oxygen, organic substrates, or other amendments (Tyagi et al., 2011). Biostimulation has been widely used for the remediation of petroleum hydrocarbons, chlorinated solvents, and other organic pollutants. The method is especially appealing because it uses microbes that are naturally occurring and local to the environment. The benefits of over-fertilization can be seen; however, it can also cause an imbalance in the ecosystem or unintended environmental effects. So, it is important to carefully assess and monitor the sites to ensure successful implementation (Bento et al., 2005).

### 6.3 Bioaugmentation

**FIGURE 13:**
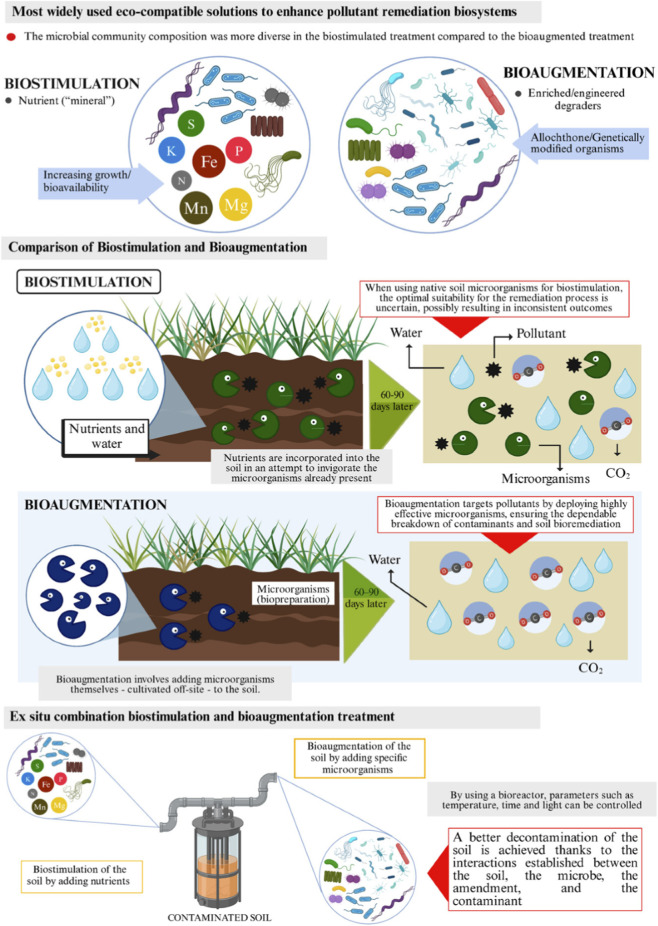
Comparison of biostimulation and bioaugmentation strategies for enhancing microbial remediation of contaminated soils (Perez-Vazquez et al., 2024).

Bioaugmentation is the process of adding specific microorganisms to polluted environments to promote decomposition of pollutants. Such microorganisms can be naturally occurring, which have been enriched for their ability to degrade, microbial consortia, or genetically optimized with specific degradative properties. The technology is especially beneficial in cases where native microbes cannot degrade target contaminants effectively or degradation rates are not high enough. Bioaugmentation has successfully been used to address petroleum hydrocarbons, chlorinated solvents, pesticides, dyes, and pharmaceutical contaminants. The survival of microorganisms, competition with native microorganisms, environmental compatibility, contaminant bioavailability, and nutrient availability are all crucial factors that affect the success of the bioaugmentation process (Gentry et al., 2004). The microorganisms introduced into the contaminated environment should be able to become active in the environment, and should remain active enough to meet the remediation goal. There have been recent developments in the field of microbial ecology and microbial biotechnology, which have facilitated the development of customized microbial consortia to be used on a site-specific basis for remediation (Cycoń, 2026). Figure 13 compares biostimulation and bioaugmentation approaches, illustrating how nutrient addition or specialized microorganisms can enhance pollutant degradation and improve bioremediation efficiency.

### 6.4 Rhizoremediation

**FIGURE 14:**
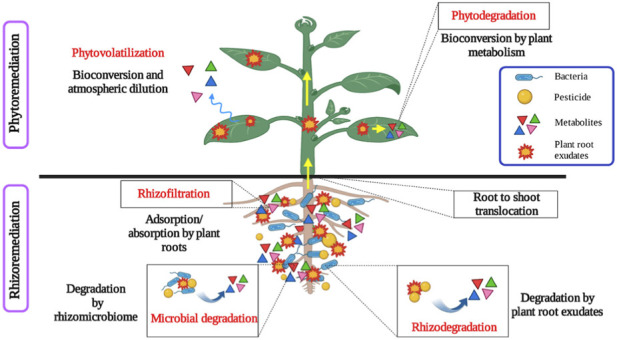
Schematic illustration of *in situ* phytoremediation and *ex situ* bioreactor-based bioremediation techniques for contaminant removal (Perez-Vazquez et al., 2024).

Rhizoremediation is one of the technologies in the category of plant-microbe-assisted remediation that involves interactions between plant roots and rhizosphere microorganisms to improve the removal of contaminants from soil. Organic compounds like sugars, amino acids, and organic acids are excreted by plants and stimulate microbial growth and activity in the rhizosphere, which creates a nutrient-rich environment (Gerhardt et al., 2009). This is followed by the degradation, transformation or immobilisation of contaminants in the soil surrounding the rhizosphere by its microorganisms. It has proven to be effective for the remediation of petroleum hydrocarbons, PAHs, pesticides, chlorinated compounds and some heavy metals. Grasses, legumes and fast-growing woody plants are examples of common plants that can be used for rhizoremediation (Kuiper et al., 2004). The benefits of rhizoremediation are few: low cost, limited disturbance of the environment, improved soil structure, enhanced ecosystem restoration. In Figure 14 summarizes plant–microbe interactions in phytoremediation and the use of bioreactors for *ex situ* microbial degradation of contaminants, highlighting complementary approaches for environmental remediation.

### 6.5 Mycoremediation

Mycoremediation is the use of fungi as a means of remediation of contaminated environments. Fungi have large hyphal networks and potent extracellular enzyme systems which are able to break down complex organic pollutants which are frequently not metabolized by bacteria. White-rot fungi have been the subject of much interest because of the generation of ligninolytic enzymes like laccase, lignin peroxidase, and manganese peroxidase (Pointing, 2001). These enzymes are highly promiscuous and can break down PAHs, dyes, pesticides, pharmaceuticals, and chlorinated organic compounds. Fungi can also play a role in heavy metal removal *via* biosorption, bioaccumulation and immobilization processes in addition to organic contaminant degradation. They can also remediate poorly available contaminants by invading soil matrices. Mycoremediation is beginning to be recognized as a sustainable and versatile technology for treatment of complex contaminant mixtures (Harms et al., 2011).

### 6.6 Microbially induced carbonate precipitation (MICP)

Microbial Induced Carbonate Precipitation (MICP) is an established bio-geotechnical approach that has gained considerable attention for soil improvement and contaminant immobilization applications. It is typically accomplished by ureolytic bacteria such as Sporosarcina pasteurii, which decompose the urea and generate carbonate ions that interact with the calcium ions to generate calcium carbonate precipitates (Achal et al., 2011). The first uses of MICP have been to stabilize and improve the ground, and recently, there have been projects underway to remediate the environment. Heavy metals and metalloids can be immobilized by calcium carbonate precipitation *via* adsorption, co-precipitation, and encapsulation mechanisms, decreasing contaminant mobility and bioavailability. MICP has several benefits such as being environmentally friendly, requiring relatively low energy, and being able to be used for both soil improvement and contaminant stabilization (Cheng et al., 2013). While ureolysis-based MICP is one of the extensively studied pathways, there are various restrictions associated with it, which include the formation of ammonia as a byproduct in the case of urea hydrolysis, external source requirements of urea and calcium, pH changes, heterogeneous precipitation of calcite, and clogging of pores. Moreover, the permanence of metal stabilization might differ depending on environmental conditions. The use of other types of carbonate precipitation processes such as denitrification and photosynthesis-based reactions is considered a solution to these restrictions (Omoregie et al., 2024). In recent years, significant progress has been made to expand the application of MICP in geo-environmental engineering. In addition to soil stabilization, MICP has been investigated for heavy metal immobilization, groundwater remediation, erosion protection, crack sealing in construction materials, and contaminated site remediation. Also, it has been coupled with bioaugmentation and nutrient optimization processes to enhance the efficiency of calcite precipitation under different environmental conditions. It has been shown to be effective at improving the integrity of soils and decreasing movement of contaminants, with minimal impact on the environment when compared to traditional chemical stabilization techniques, in pilot-scale studies. However, issues of large-scale implementation, long-term sustainability, ammonia management and process optimization are still active research areas, highlighting the need to further test in the field and develop sustainable non-ureolytic pathways.

Although laboratory tests were successful, large scale implementation of MICP is still difficult because of several problems including the ammonia production, the non-uniform calcium carbonate precipitate distribution, and the long-term field performance, which needs to be studied further.

### 6.7 Permeable reactive biobarriers

Permeable Reactive BioBarriers (PRBs) are subsurface treatment barriers that capture and treat contaminated groundwater plumes. These barriers are placed in the flow path of the contaminant, and they include materials that are reactive or microbial communities that are capable of removing the contaminant. The microbial biobarriers (Microbial Biofilters) are a type of treatment system that uses naturally occurring or introduced microorganisms to break down organic contaminants as contaminated groundwater flows through (Obiri-Nyarko et al., 2014). The barriers can facilitate both aerobic and anaerobic microbial processes. PRBs are effective in the clean-up of petroleum hydrocarbons, chlorinated solvents, nitrate contamination, and other industrial contaminants. Passive treatment requires minimal excavation and site disturbance, with long treatment times (Gavaskar, 1999). While there are significant environmental and economic benefits of using PRBs, performance over time depends on maintaining the PRB in a state of hydraulic conductivity, microbial activity, and reactivity. The recent development of permeable reactive biobarriers has led to their use for the treatment of complex mixtures of organic and inorganic contaminants, in addition to their use in groundwater treatment. In modern PRBs, the microbial consortium is often supplemented with reactive materials like activated carbon, biochar, zero-valent iron, or organic materials as substrates to promote degradation and transformation of the contaminants. Studies have shown effective removal of emerging contaminants, nitrates, chlorinated solvents, and petroleum hydrocarbons without sacrificing passive, low-energy operation, at the field and pilot scales. But over time, these can lead to media clogging, lower permeability, substrate depletion, and changes in the composition of the microbial community. Current research is directed towards enhancing barrier life, maximizing microbial activity, and creating hybrid PRB systems for long-term *in situ* remediation. Table 1 shows the major microbial remediation technologies used in geo-environmental engineering. The following table highlights their principles, contaminants targeted, and major benefits to sustainable remediation.

**TABLE 1:** Major microbial technologies used in geo-environmental engineering remediation.

Technology	Principle	Target pollutants	Key advantages	Main limitations	Process efficiency	Remediation time	References
Natural Attenuation	Utilizes indigenous microorganisms and natural processes for contaminant reduction	Petroleum hydrocarbons, chlorinated solvents, pesticides	Low cost, minimal disturbance, environmentally friendly	Slow treatment, strong dependence on site conditions, uncertain performance for persistent or mixed contaminants, requires long term monitoring and evidence of plume stability	Moderate	Months-years	Mishra et al. (2025)
Biostimulation	Addition of nutrients/electron donors to stimulate native microbes	Petroleum hydrocarbons, chlorinated solvents, organic pollutants	Enhances indigenous microbial activity	Overdosing may alter redox conditions, cause nutrient imbalance, mobilize some contaminants, or stimulate non-target microbial processes. Field performance depends on amendment delivery and contaminant bioavailability	High	Weeks-months	Tyagi et al. (2011)
Bioaugmentation	Introduction of specialized degradative microorganisms	Hydrocarbons, pesticides, dyes, and pharmaceuticals	Site-specific and rapid degradation	Introduced microorganisms may fail to persist because of competition, predation, poor adaptation, contaminant toxicity, or unfavorable soil conditions. Performance requires verification against non-inoculated controls	High	Weeks-months	Tyagi et al. (2011)
Rhizoremediation	Plant–microbe interactions in the rhizosphere enhance degradation	PAHs, hydrocarbons, pesticides, heavy metals	Sustainable, improves soil quality	Limited root depth, seasonal plant activity, contaminant phytotoxicity, variable plant-microbe interactions, and possible transfer of contaminants into plant biomass	Moderate	Months	Gerhardt et al. (2009)
Mycoremediation	Fungal degradation using extracellular enzymes	PAHs, dyes, pesticides, pharmaceuticals	Effective for complex pollutants	Sensitive to moisture, pH, temperature, aeration, and contaminant toxicity. Field establishment may be difficult, and transformation products require monitoring	High	Weeks	Harms et al. (2011)
MICP	Microbial precipitation of CaCO_3_ for stabilization and immobilization	Heavy metals, metalloids	Soil improvement, contaminant immobilization	Ureolytic MICP can generate ammonium, requires calcium and substrate delivery, may cause clogging, produces heterogeneous precipitation, and may lose immobilization stability under changing pH or redox conditions	High	Days-weeks	Cheng et al. (2013)
Permeable Reactive Biobarriers (PRBs)	Microbial treatment within subsurface reactive barriers	Groundwater hydrocarbons, chlorinated solvents, nitrates	Passive long-term remediation	Performance may decline because of media clogging, permeability loss, substrate depletion, changes in microbial activity, and heterogeneous groundwater flow	High	Months	Obiri-Nyarko et al. (2014)
Bioelectrochemical Remediation Systems	Utilizes electroactive microorganisms and microbe-electrode interactions to degrade or transform contaminants	Petroleum hydrocarbons, dyes, halogenated compounds, and selected heavy metals	Sustainable remediation, enhanced electron transfer, and potential energy recovery	Limited by electrode cost, fouling, low mass transfer in soil, electrode spacing, conductivity, moisture control, scale up constraints, and the need to maintain active electroactive biofilms	Moderate to High	Weeks-Months	Logan and Rabaey (2012)

### 6.8 Bioelectrochemical remediation systems

The bioelectrochemical systems (BES), such as the microbial fuel cell and microbial electrolysis cell, take advantage of electroactive microbes to promote the decomposition or transformation of contaminants *via* microbe-electrode reactions. These types of biotechnologies have been explored for the decontamination of petroleum hydrocarbons, dyes, halogenated substances, and some heavy metals by means of control of redox processes. BES operation is based on various parameters like electrodes, microbial population, substrate presence, and environmental variables. Even though these technologies promise to offer sustainable decontamination and even electricity generation, limitations such as high initial cost, scaling issues, and fouling hinder large-scale applications (Logan and Rabaey, 2012).

## 7 Factors affecting remediation efficiency

Many physical, chemical, biological, and environmental factors will affect the success of microbial remediation. Microorganisms can break down, transform, or immobilize contaminants, but site-specific conditions can affect the efficiency of remediation. The availability of contaminants, microbial activity, and remediation performance depend on soil characteristics, environmental conditions, pollutant properties, and microbial community dynamics. Such knowledge is critical for making effective remediation designs and for designing optimal application of geo-environmental engineering systems at the field-scale. Table 2 summarizes key microbial factors that affect the efficiency of remediation.

**TABLE 2:** Factors affecting microbial remediation efficiency.

Factor	Major components	Effect on remediation	References
Soil Properties	Texture, pH, porosity, organic matter, CEC	Controls microbial activity and contaminant bioavailability	Semple et al. (2003)
Environmental Conditions	Temperature, moisture, oxygen, nutrients, redox conditions	Influences microbial metabolism and degradation rates	Megharaj et al. (2011)
Pollutant Characteristics	Structure, solubility, toxicity, concentration	Determines biodegradability and accessibility	Haritash and Kaushik (2009)
Microbial Factors	Diversity, functional genes, biofilms, adaptation	Controls degradation pathways and resilience	Flemming et al. (2016)

### 7.1 Soil properties

The properties of soil are important factors in controlling bioavailable contaminants and microbial activity and behavior. Soil physical and chemical properties affect the growth of soil microorganisms, nutrient availability, mobility of contaminants, and degradation efficiency. The media composition of soil has a strong influence on remediation activities. Soils that are sandy allow for high permeability and oxygen diffusion, and promote microbial activity, leading to contamination transport (Alexander, 1999). On the other hand, the permeability and adsorption capacity of clay-rich soils are smaller and greater, respectively, than other soils and result in lower levels of contaminants and microbial activity. The movement of water through a soil, the amount of soil water, and the movement of nutrients and contaminants in the soil will be influenced by the structure and porosity of a soil. Microbial metabolism and enzymatic activity are also influenced by soil pH. Most of the microorganisms that degrade pollutants thrive under neutral or slightly alkaline soil conditions, while soils with high acidity or alkalinity may harm the microorganisms and lower the rate of degradation. Additionally, the remediation efficiency is affected by the organic matter content due to its role as a nutrient source and its effects on the adsorption behaviour of the contaminants (Semple et al.). The effectiveness of microbial remediation is also enhanced by cation exchange capacity, the ability of soils to retain moisture, and the soil mineral content. Thus, soil characterization is crucial before adoption of remediation technologies.

### 7.2 Environmental conditions

Microbial survival, metabolism, and contaminant degradation processes are greatly affected by environmental conditions. The efficiency of microbial remediation systems depends on factors like temperature, moisture content, oxygen, nutrients, and redox conditions. Enzymatic activity and growth rates of microorganisms are affected by temperature. The majority of soil microorganisms capable of bioremediation have mesophilic temperature ranges. Very cold temperatures slow down metabolic activity, and very hot temperatures can denature enzymes and inhibit microbial growth (Megharaj et al., 2011). Moisture content is also significant, as the availability of moisture ensures the transport of nutrients within the microbial system, and metabolic reactions take place. Water-deficient and water-saturated conditions can both have a negative effect on remediation performance. Too much water can hinder the flow of oxygen, and too little can slow microbes down. Availability of oxygen is critical in aerobic degradation pathways. Numerous microorganisms are able to break down hydrocarbons in the presence of oxygen as a terminal electron acceptor. Under anaerobic conditions, the electron acceptors other than oxygen that are important to microbial metabolism are nitrate, sulfate, ferric iron, and carbon dioxide. Remediation efficiency is also affected by the availability of essential nutrients, especially nitrogen and phosphorus t. Nutrient limitations can often limit the growth of microbes and degradation of pollutants, so nutrient supplementation is frequently included in biostimulation approaches.

### 7.3 Pollutant characteristics

Physicochemical properties of contaminants are of great importance in their ability to be remediated by microbes. The chemical structure, concentration, solubility, toxicity, and bioavailability of pollutants determine the ability of microorganisms to gain access and metabolize the pollutants. An important factor in determining biodegradability is the chemical structure. Simple organic compounds will be more easily degraded than highly complex compounds. Pollutants with aromatic rings, halogen substitutions, and high MWs are more persistent and microbial degradation-resistant. One of the factors that is important for remediation efficiency is the bioavailability (Poblete-Castro et al., 2012). Most of the hydrophobic contaminants are tightly absorbed on soil particles and organics, which limits the microorganisms’ access. However, in the presence of microorganisms that degrade the contaminant, remediation can be limited due to poor availability of the contaminant. Microbial activity is also affected by the concentration of contaminants. Moderate concentrations can stimulate microbial degradation and adaptation, whereas high concentrations may be toxic to the microbial populations and detrimental to microbial remediation processes. Mixed contamination scenarios where interactions between multiple contaminants can lead to stimulation and/or inhibition of microbial degradation pathways will further complicate remediation.

### 7.4 Microbial factors

The effectiveness of remediation strategies is greatly affected by microbial characteristics. Contaminant transformation efficiency is determined by diversity and abundance of microbial communities, the presence of functional degradative genes, biofilm-forming ability, and adaptation to environmental stress (Flemming and Wingender, 2010), (Top and Springael, 2003). Also, the interactions between introduced and native microorganisms may impact the long-term effectiveness of bioaugmentation.

## 8 Monitoring and characterization techniques

The performance of microbial remediation processes in contaminated soil and geo-environmental systems requires effective monitoring and characterization. Accurate analysis methods can be used to help researchers analyze contaminant degradation, track changes in the microbial community, measure the effectiveness of remediation, and assess impacts on the environment. Over recent years, the knowledge of microbial remediation processes has been greatly enhanced thanks to the development of molecular biology, high-throughput sequencing, omics approaches, and sensor-based monitoring. Monitoring methods should be associated with specific endpoints for remediation. Parent organic contaminants and transformation products are often quantified by gas chromatography (GC) and gas chromatography–mass spectrometry (GC–MS) while less volatile organic compounds are often analysed using high-performance liquid chromatography (HPLC). Combined measurements of parent compounds, intermediate metabolites and mineralization products may be necessary for total contaminant removal and mass balance assessments. Evaluating for residual toxicity can be done with ecotoxicological assays and bioassays to assess if remediation has lessened biological effects. Heavy metals and metalloids are tested for total concentration, metal speciation and bioavailability using such methods as ICP–MS, X-ray spectroscopy and sequential extraction procedures. The geochemical stability is typically assessed by monitoring pH, redox potential, dissolved oxygen, and other environmental factors that affect the mobility and long-term stability of the contaminants. Moreover, molecular biology methods such as quantitative PCR, metagenomics, and transcriptomics can be used to measure the activity and composition of microbial communities to assess if the microorganisms that are responsible for contaminant degradation and transformation are active during remediation. No single indicator should be used to declare microbial remediation successful. Parent compound reduction is insufficient when toxic transformation products persist, metal mobility increases, contaminant bioavailability remains high, plume stability is not demonstrated, or microbial functional activity declines (Belezia and Almeida, 2021). Remediation performance should be assessed by a weight-of-evidence framework that integrates contaminant concentrations, transformation products, mineralization indicators, toxicity tests, bioavailability measurements, geochemical stability, groundwater plume behavior, and microbial activity. When indicators conflict, the conclusion should follow the more conservative risk interpretation until residual hazard has been ruled out. Analytical data should also be supported by quality assurance and quality control procedures, including method validation, limits of detection, limits of quantification, analyte recovery, matrix-effect assessment, calibration standards, procedural blanks, certified reference materials when available, and replicate measurements. These procedures improve accuracy, precision, and comparability of the data used to judge remediation outcomes (EPA, 2002).

### 8.1 Conventional analytical methods

Traditional analytical techniques are still important tools used to characterize contaminated sites and to assess the effectiveness of remediation. These techniques are mainly used for the detection of contaminants, measurement of pollutant levels, assessment of degradation rates, and analysis of soil properties changes during the remediation process. Volatile and semi-volatile organic pollutants such as petroleum hydrocarbons, polycyclic aromatic hydrocarbons (PAHs), pesticides, and chlorinated compounds are commonly detected and quantified using Gas Chromatography (GC) and Gas Chromatography–Mass Spectrometry (GC–MS) (Csuros and Csuros, 2016). Pharmaceuticals, antibiotics, dyes, and other emerging contaminants can be analysed using High-Performance Liquid Chromatography (HPLC) and Liquid Chromatography-Mass Spectrometry (LC-MS). Heavy metal analysis can be performed with highly sensitive quantification of metal concentrations, for example, Atomic Absorption Spectroscopy (AAS), Inductively Coupled Plasma Optical Emission Spectroscopy (ICP-OES) and Inductively Coupled Plasma Mass Spectrometry (ICP-MS). Other soil characteristics such as pH, organic matter content, moisture content, redox potential, and electrical conductivity are also routinely measured to assess the conditions affecting microbial processes. While these traditional techniques yield valuable data on contaminant fate and remediation effectiveness, they fail to directly elucidate the structure of the microbial community and its metabolic processes (Hurst et al., 2007). The physicochemical parameters of the contaminated environment are highly influential in the microbial remediation process. Microbial growth, enzyme activity, and pollutant bioavailability are affected by factors including soil pH, temperature, moisture, dissolved oxygen, nutrients, contaminants, and soil organic matter. Therefore, it is important to keep these parameters within appropriate ranges to obtain optimum biodegradation efficiencies and good remediation results. The optimal ranges of these parameters and what effect they will have if the parameters deviate from the recommended values are summarized in Table 3.

**TABLE 3:** Environmental parameters influencing effective microbial remediation in soil and geo-environmental systems.

Parameter	Primary matrix	Typical target condition or interpretive range	Effect below target condition	Effect above target condition	Main interpretation for remediation assessment	References
pH	Soil, sediment, groundwater	Commonly near neutral to slightly alkaline, approximately 6.5 to 8.0 for many bacterial degradation processes	Acidic conditions may reduce microbial growth, enzyme activity, nutrient availability, and degradation rates	Strongly alkaline conditions may inhibit microbial activity and alter metal mobility, sorption, and contaminant speciation	Interpret with contaminant chemistry, microbial group, and target process. Metal and metalloid remediation requires speciation-based interpretation, not pH alone	Vidali (2001)
Temperature	Soil, sediment, groundwater	Commonly 20 °C–35 °C for mesophilic microbial processes	Low temperature slows enzymatic activity, microbial growth, contaminant transformation, and mass transfer	High temperature may inhibit microbial growth, denature enzymes, or shift microbial community composition	Report site temperature during treatment. Temperature effects should be interpreted with moisture, oxygen, nutrient supply, and microbial activity	Megharaj et al. (2011)
Moisture content	Unsaturated soil, vadose zone soil, sediment	Sufficient moisture for microbial metabolism and nutrient diffusion, commonly expressed as percent water holding capacity	Low moisture restricts nutrient transport, substrate diffusion, microbial activity, and enzymatic reactions	Excess moisture can limit oxygen diffusion, create anaerobic microsites, and shift redox conditions	Report as gravimetric water content, volumetric water content, or percent water holding capacity. Do not compare directly with dissolved oxygen values from groundwater	Vidali (2001)
Dissolved oxygen	Groundwater, porewater, saturated soil, slurry systems	Process specific. Aerobic hydrocarbon and many PAH degradation processes generally require measurable dissolved oxygen	Oxygen limitation can slow aerobic degradation of hydrocarbons, PAHs, and some pesticides	High oxygen may provide limited additional benefit for aerobic processes and may inhibit strictly anaerobic reductive processes	Report as mg L^-1^ only for aqueous phases, including groundwater and porewater. For unsaturated soil, report oxygen availability through gas phase oxygen, redox conditions, or aeration status	Das and Chandran (2011)
Redox condition	Groundwater, sediment, saturated soil, periodically saturated soil	Process specific, depending on whether aerobic oxidation, nitrate reduction, sulfate reduction, iron reduction, methanogenesis, or reductive dechlorination is targeted	Redox conditions that are too reducing may limit aerobic degradation of hydrocarbons and many PAHs	Redox conditions that are too oxidizing may limit reductive dechlorination and some metal or metalloid transformations	Interpret with electron acceptors, electron donors, contaminant class, and transformation products. Redox status should not be inferred from dissolved oxygen alone	Megharaj et al. (2011)
Nutrient availability, especially C:N:P balance	Soil, sediment, groundwater, biostimulated systems	Site specific. A commonly used starting ratio for hydrocarbon biostimulation is approximately 100:10:1 for C:N:P	Nitrogen or phosphorus limitation may restrict microbial growth, enzyme production, and contaminant degradation	Excess nutrients may cause microbial imbalance, secondary pollution, oxygen depletion, or unwanted redox shifts	Nutrient amendment should be justified by site testing. Report amendment type, dose, delivery method, and monitoring response	Tyagi et al. (2011)
Hydrocarbon concentration	Soil, sediment, groundwater contaminated by petroleum hydrocarbons	No universal optimum. Concentration should be below levels that cause strong microbial toxicity and high enough for measurable degradation assessment	Very low concentrations may limit enrichment of hydrocarbon degraders and reduce measurable degradation rates	High concentrations may be toxic to microbial cells, reduce oxygen transfer, increase hydrophobic phase persistence, and decrease bioavailability	Report hydrocarbon fraction, initial concentration, analytical method, and endpoint. Parent compound decline should not be equated with mineralization unless mineralization was measured	Varjani (2017)
Heavy metal or metalloid concentration	Soil, sediment, groundwater, mixed organic-inorganic contamination	No universal optimum. Acceptable levels depend on element, speciation, pH, redox status, and bioavailability	Low concentrations may cause limited direct inhibition but can still require risk assessment if toxicity thresholds are exceeded	Elevated concentrations may inhibit microbial growth, enzyme activity, organic contaminant degradation, and community stability	Report total concentration together with speciation, mobility, bioavailability, and toxicity. Transformation alone does not prove risk reduction	Ojuederie and Babalola (2017)
Soil organic matter	Soil, sediment	Site specific. Moderate organic matter may support microbial habitat and nutrient supply	Low organic matter may limit microbial biomass, nutrient retention, and soil structure	High organic matter can increase sorption of hydrophobic contaminants and reduce bioavailability to microorganisms	Interpret with contaminant hydrophobicity, aging, sorption strength, and desorption kinetics. Organic matter can support microbes while reducing contaminant accessibility	(Semple et al.)
Electrical conductivity and salinity	Soil, sediment, groundwater	Site specific. Should remain within the tolerance range of the active microbial community	Very low ionic strength may affect nutrient availability and microbial activity in some systems	High salinity or conductivity may impose osmotic stress, inhibit sensitive taxa, and alter metal mobility	Report with pH and redox data. Interpret salinity effects with microbial tolerance, contaminant chemistry, and amendment history	(Vidali, 2001)

### 8.2 Molecular techniques

Microbial communities in environmental remediation have undergone a revolution with molecular techniques. Moreover, molecular tools facilitate the identification and characterization of culturable and non-culturable microorganisms by analyzing environmental samples, whereas culture techniques do not enable such an analysis. Specific genes and species of microbes that can break down pollutants can be targeted using the Polymerase Chain Reaction (PCR). Furthermore, quantitative PCR (qPCR) is being used to quantify specific target genes, which are part of degradation pathways and help to provide information regarding the amount of microbes involved in bioremediation processes (Amann et al., 1995). Fluorescence *in situ* hybridization (FISH) can be used to detect and locate microorganisms in soil. The other common techniques utilized for the profiling of the microbial community in bioremediation studies include denaturing gradient gel electrophoresis (DGGE) and terminal restriction fragment length polymorphism (T-RFLP). The emergence of next-generation sequencing (NGS) has helped researchers to understand microbial communities better. For example, bacterial populations may be characterized through the sequencing of 16S rRNA genes, while ITS gene sequences can be used to identify fungal populations (Muyzer et al., 1993). It is important that microbial bioremediation assessment is not limited to changes in concentrations of parent compounds only but must include all four groups of criteria: chemical criteria, which can include changes in contamination concentrations, formation of metabolites, mass balance; biological criteria, which include microbial biomass and functional activity; ecotoxicological criteria, which cover residual contamination toxicity and associated risk to ecosystems; and geochemical criteria, which include metal speciation, availability of elements and immobility of contaminants. Assessment of microbial bioremediation should not only include changes to parent compound concentrations, but also include the formation of the metabolites, mass balance, microbial mass and activity, residual toxicity, metal speciation, contaminant bioavailability, and the stability of the plume if applicable. These complementary chemical, biological, ecotoxicological and geochemical criteria allow a more holistic assessment of remediation success. Analytical data also must be accompanied by suitable quality assurance and quality control (QA/QC) protocols, such as method validation, limits of detection (LOD), analyte recovery, evaluation of matrix effects, procedural blank, calibration standard, and replicate testing.

### 8.3 Omics approaches

Omics technologies have turned out to be effective tools for studying the roles and interactions of microbes in the process of remediation. This approach provides an analysis of genes, transcripts, proteins, and metabolites of microbes involved in the biodegradation of the contaminant. Metagenomics can be used to study the total metagenome found in the environmental sample, and without growing the microorganisms, the researchers will be able to get information on genes and metabolic pathways that play a significant role in the process of degradation (Jansson and Hofmockel, 2020). Metatranscriptomics analyzes expressed genes within an active microbial community and gives information about how the microbial community behaves in response to the environment and contamination. Other omics approaches include metaproteomics, which analyses and quantifies proteins associated with the microbial community and its role in the degradation of pollutants, and metabolomics for studying the intermediate products of the metabolic reactions. This data can aid in the development of optimized remediation strategies and engineered microbial consortia. Although the omics technologies have many benefits, they tend to be complex and require high-level instrumentation, bioinformatics skills, and computational power (Tyc et al., 2017).

### 8.4 Biosensors and real-time monitoring

In recent years, the development of biosensor technology has allowed the monitoring in real time, rapidly and sensitively, of contaminants and microbial activity in environmental systems. The biosensors combine the biological recognition elements and the signal transduction system in order to detect specific analytes and environmental changes. Microbial biosensors are living microorganisms that are genetically engineered and biologically selected to produce measurable biological signals when exposed to specific contaminants (Rodriguez-Mozaz et al., 2006). DNA-based biosensors are used for target genes or microbial biomarkers related to the remediation process, and enzyme-based biosensors are used for detecting pollutants through catalytic reactions. Electrochemical biosensors are receiving a lot of attention because of their highly sensitive, portable, and field-deployable features. The monitoring capabilities have been further enhanced by the use of optical biosensors, fluorescence-based sensors, and by the use of some kind of platform-based technology that uses some nanomaterial. Monitoring techniques that provide “real-time” information on the amount of contamination, biological activity and other environmental parameters will help make remediation efforts easier to manage. Furthermore, the combination of biosensors and wireless technologies, AI and IoT systems will significantly change the way environmental monitoring will be done in the future. While many of the benefits of biosensors are apparent, such as their speed and low cost, some issues need to be addressed in terms of their stability, calibration, and large-scale application in the field (Turner, 2013).

## 9 Challenges and limitations

While considerable advancements have been made in the field of microbial remediation techniques, several microbial, technical, and practical issues continue to pose obstacles to effective implementation at scale. While laboratory-scale testing has yielded some positive results, it is not an easy task to apply this approach on a field scale owing to various environmental complexities. One of the major obstacles is the issue of the bioavailability of various recalcitrant pollutants. Hydrophobic pollutants such as PAHs, petroleum hydrocarbons, and chlorinated organic compounds have a high affinity for soils and organic matter, reducing their microbial accessibility and slowing down the degradation process. In the same manner, toxic pollutants that are very resistant to degradation, such as PFAS, are extremely challenging to remediate *via* complete microbial degradation due to their high resistance against both physical and biological processes (Azubuike et al., 2016). Ideal conditions that could enable laboratory research are not easy to obtain under field conditions. The main problem with the process is the viability of introduced microorganisms when undertaking bioaugmentation. This might be affected by other microbial populations or predation and environmental stress. In addition, mixed contaminant systems usually require several pathways for degradation as opposed to single contaminants. Remediation monitoring is yet another problem associated with the process. Although new methods based on molecular science and omics technologies have made it possible to understand microbial communities, establishing a link between microbial action and contaminant reduction remains difficult (Harms et al., 2011). Moreover, if we use advanced analytical tools, we should have some special skills and a significant amount of money. Acceptability and regulatory concerns also have an impact on the application of microbial remediation technologies, especially if chemically engineered microorganisms are used. Care must be taken in evaluating the long-term environmental impacts, ecological risks, and biosafety issues before large-scale use. Therefore, addressing these challenges in science and engineering is critical to improving the reliability, scalability and sustainability of microbial remediation technologies (Lea-Smith et al., 2025).

## 10 Emerging trends and future perspectives

Recent advances in environmental biotechnology, microbial ecology, synthetic biology, data science, and materials engineering are opening new opportunities to enhance microbial systems for remediation. Future studies will look to increase remediation efficiency, increase the number of contaminants that can be degraded, and increase the monitoring capability, as well as combine biological processes with sustainable engineering solutions. Future research should move beyond proof-of-concept laboratory studies toward hypothesis-driven field validation. Key research questions include whether engineered microbial consortia can maintain long-term stability and degradation performance under fluctuating environmental conditions, how microbial community composition changes during extended remediation periods, and whether multi-species systems consistently outperform indigenous microbial populations under field conditions. Future validation studies should include pilot-scale and field-scale trials with appropriate untreated controls, long-term monitoring programs, and standardized performance metrics. Monitoring should evaluate not only contaminant removal but also transformation products, mineralization, residual toxicity, contaminant bioavailability, geochemical stability, groundwater plume behavior, and microbial functional activity. The deployment of engineered microorganisms and synthetic biology approaches should be accompanied by comprehensive biosafety assessments addressing ecological impacts, horizontal gene transfer, persistence of introduced microorganisms, and potential unintended environmental consequences. Multi-omics technologies should be further developed to identify functional genes, metabolic pathways, and microbial interactions that control contaminant degradation under field conditions. Artificial intelligence and machine learning tools may provide practical support for predictive modeling, process optimization, contaminant transport forecasting, and remediation decision-making when integrated with environmental monitoring datasets. These technologies are likely to be most effective in complex contaminated sites where multiple contaminants, heterogeneous environmental conditions, and long remediation periods challenge conventional remediation strategies.

### 10.1 Synthetic biology

**FIGURE 15:**
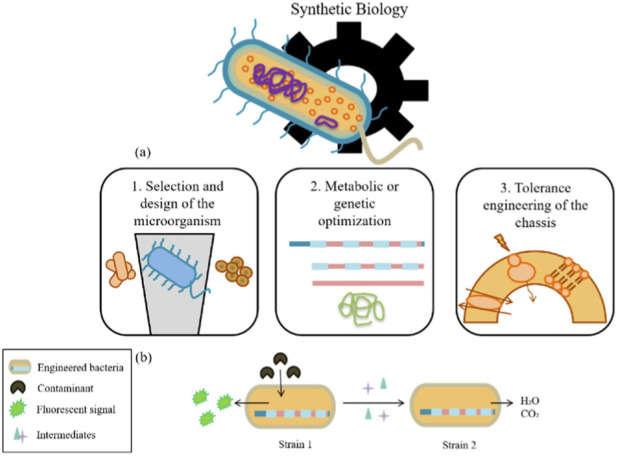
Synthetic biology approaches for enhanced microbial bioremediation of environmental pollutants (Jiménez-Díaz et al., 2022).

Synthetic biology has become an exciting new area with great promise in environmental remediation. Genetic engineering and metabolic pathway optimization techniques can be used to genetically engineer microorganisms to degrade pollutants that are naturally resistant to degradation. Synthetic biology can be used to introduce novel catabolic pathways, to increase the production of enzymes, and to increase the tolerance of microbial stress (Sayler and Ripp, 2000). Engineered microorganisms can be created to remove specific contaminants, such as chlorinated compounds, pharmaceuticals, and PFAS. Synthetic gene circuits also have the potential to enhance environmental sensing and pollutant-responsive degradation systems. While significant advances have been made, practical use of engineered microorganisms for environmental applications also has to take into account biosafety issues and regulatory hurdles (Das et al., 2025). Figure 15 illustrates the role of synthetic biology in advancing microbial remediation through engineered microorganisms, metabolic pathway optimization, and synthetic microbial consortia. These strategies improve pollutant detection, degradation efficiency, and microbial tolerance, providing promising solutions for the remediation of recalcitrant environmental contaminants.

### 10.2 Engineered microbial consortia

Complex microbial communities may be responsible for natural remediation processes instead of individual species. Thus, the design of engineered microbial consortia is currently under intense research. Engineered consortia are groupings of microorganisms that have complementary metabolic functions to provide for more complete degradation of many complex contaminant mixtures (Renganathan et al., 2025). These systems can help make the most of substrate oxidation, boost tolerance to environmental stress, and allow for full mineralisation of pollutants by following metabolic pathways. Recent breakthroughs in systems biology, metabolic modelling, and microbial ecology are allowing microbial communities to be rationally designed to meet specific remediation goals. These methods are supposed to enhance the efficiency and adaptability of remediation in the field (Lindemann et al., 2016). Engineered microbial consortia are superior degraders of complex mixtures of organic pollutants compared to single microbial strains, as they possess greater degradation efficiencies than single microorganisms, which can be achieved by synergistic metabolic interactions and division of labor among the microbes.

### 10.3 Nanobioremediation

Nanobioremediation uses nanotechnology to complement biological remediation processes and improve contaminant removal and monitoring. Nanomaterials can increase pollutant bioavailability, enhance microbial growth, promote electron transfer, and enhance degradation. There are several nanomaterials, such as metal nanoparticles, carbon-based nanomaterials, biochar nanocomposites, and magnetic nanoparticles, that have been studied for remediation (Kumar et al., 2026). These materials can be used as an adsorbent, a catalyst, a nutrient carrier, or a matrix for microbial support. Nanotechnology and microbiology have been integrated and are promising for the degradation of hydrocarbons, pesticides, dyes, pharmaceuticals, and heavy metals. The possibility of environmental hazards due to the release of nanoparticles, however, needs to be investigated further (Karn et al., 2009). Nanomaterial-assisted microbial systems, such as biochar-based composites and metal nanoparticles, have been reported in recent studies to improve the bioavailability and microbial activity for enhanced degradation of hydrocarbons, dyes, and pharmaceutical contaminants. Recent studies have pointed to the high efficiency of engineered microbial consortia in degradation, but problems of community stability, ecological interactions, and deployment on field scale still prevent the large-scale use of these consortia.

### 10.4 AI and machine learning

AI and ML should be presented as decision-support tools, not as substitutes for chemical monitoring or field validation. Useful models require curated data that include contaminant identity and concentration, transformation products, soil texture, organic matter, pH, moisture, redox potential, temperature, nutrient status, electron acceptors, microbial community profiles, functional genes, treatment design, and time-resolved remediation outcomes. Model development should report data provenance, preprocessing, feature selection, training and test separation, uncertainty analysis, and external validation on sites not used for model fitting (Wang et al., 2024). Transferability across sites should be tested explicitly because soil heterogeneity, contaminant aging, mixed pollution, sparse time-series data, and unmeasured geochemical variables can cause model failure. AI is most defensible when used to rank hypotheses, identify monitoring priorities, forecast contaminant trends, or optimize operating conditions that are then verified by chemical, biological, ecotoxicological, and geochemical endpoints (Alavian and Khodabakhshi, 2025).

### 10.5 Sustainable geo-environmental applications

New remediation technologies need to fit into the concept of the sustainable future and circular economy. The demand for sustainable geoenvironmental applications is increasing, and they must include the removal of contaminants and the restoration of the environment, resources, and ecosystems (Ferreira et al., 2026). Technologies such as Bioelectrochemical systems, microbially induced carbonate precipitation (MICP), and integrated plant-microbe remediation systems can be used to achieve multiple environmental benefits. These techniques reduce risks of contamination, support soil stabilisation, reduce the risk of groundwater contamination, sequester carbon, and restore ecosystems. Microbial technologies and sustainable engineering practices are expected to contribute to the sustainability of the environment and adaptation strategies to climate change in the long term (Dejong et al., 2014). There is a need for future study of emerging microbial remediation technologies for scale-up and demonstration in a variety of environmental conditions. Long-term monitoring, biosafety assessment of engineered microorganisms, stability of microbial consortia, standardized performance evaluation protocols and the incorporation of new monitoring tools (including multi-omics and artificial intelligence) should be considered in particular. Such work will be helpful for the responsible application of research in the lab to the real environment.

## 11 Conclusion

The recalcitrant pollutants are very persistent in the environment, toxic, and not easily degraded by the natural degradation process, and are one of the big challenges in the environment. Contaminants that impact soil quality, ecosystem health, and human wellbeing, such as PAHs, petroleum hydrocarbons, chlorinated organic compounds, pesticides, pharmaceuticals, PFAS, and heavy metals, continue to pose a risk to soil. Microbial remediation has evolved as an environmentally friendly and sustainable approach that can be applied to remove, transform, immobilise, or neutralise the toxicity of these contaminants through various types of biological processes. Microorganisms responsible for remediation include bacteria, fungi, actinomycetes and microbial consortia that can perform biodegradation, biosorption, bioaccumulation, biotransformation, biomineralisation, co-metabolism and biofilm-mediated remediation. Microbial technologies have been combined with geo-environmental engineering methods such as bioaugmentation, rhizoremediation, mycoremediation, bio-stimulation, MICP and permeable reactive bio-barriers, further expanding opportunities for remediation. Although significant advances have been made, questions remain regarding environmental variability, microbial survival, pollutant bioavailability, field-scale implementation, and monitoring complexity that hinder remediation efficiencies. New technologies based on synthetic biology, microbial consortia, nano bioremediation, artificial intelligence, and advanced monitoring systems are seen as potential solutions to these constraints. There is a need for further studies on interdisciplinary research combining fields of microbiology, biotechnology, environmental engineering, data science, and sustainability. These will help to develop efficient, resilient, and environment-friendly remediation strategies to counter the current and future soil and geo-environmental pollution issues. Future research and development efforts should include field validation of microbial technologies, development of better persistence and stability of introduced microorganisms, long-term environmental performance, and development of robust monitoring systems. Biosafety issues, regulatory requirements, and standardized assessment methods are also important questions to consider prior to large scale implementation of engineered microbial systems, synthetic biology approaches, and bioelectrochemical technologies. The figures presented in this review are intended not only as visual illustrations but also as conceptual frameworks that organize current knowledge on pollutant classification, microbial degradation mechanisms, remediation technologies, monitoring strategies, and geo-environmental engineering applications. Collectively, these figures highlight the relationships among contaminants, microbial processes, environmental controls, and remediation outcomes, thereby supporting evidence-based interpretation of microbial remediation systems.
